# Candidate Variant Prioritization and Tissue-Mechanics Endpoints for Functional Validation in Scoliosis: A Computational Reanalysis

**DOI:** 10.3390/biology15212048

**Published:** 2026-11-09

**Authors:** Chaowei Zhang, Mingda Wang, Shuaiqi Yang, Chunxue Shao, Guang Yang, Ziheng Wang

**Affiliations:** 1 School of Sports Science, Lingnan Normal University, Shenchuan Avenue, Zhanjiang 524033, China; 2 Division of Computational Biology, Chinese Center of Exercise Epidemiology, Northeast Normal University, Renmin Street, Changchun 130024, China

**Keywords:** adolescent idiopathic scoliosis, bioinformatics, protein stability, structural bioinformatics, extracellular matrix, shear-wave elastography, zebrafish

## Abstract

We reanalysed published human variant records and zebrafish tissue-mechanics data to prioritize substitutions for testing and select readouts for subsequent functional assessment. We curated 121 variant records, scored 102 substitutions and retained 56 for the primary structural comparison. Four substitutions exceeded 1 kcal mol^−1^ in ThermoMPNN and EvoEF: *FLNB* L582P/Y1294C and *FNDC1* F108S/Y1880C. Targeted DynaMut2 predictions exceeded 1 for Y1294C and F108S, while Y1880C was reference-sensitive. Reanalysis of published *sspo*, *katnb1* and *vangl2* fish measurements reproduced increases in mean squared shear-wave velocity and shifts associated with N-acetyl-L-cysteine ethyl ester (NACET) treatment. We evaluated a geometry-based calibration using two untreated group means and two withheld treatment means. In 20 mutant fish measured after curve onset, velocity was associated with the most severe sagittal or coronal Cobb angle ($R^{2}\;=\;0.698$). Leave-one-animal-out root-mean-square error was 24.6° (95% internal resampling interval, 17.5°–35.0°), versus 42.3° for the training-mean baseline. The small, post-onset, mixed-plane dataset does not establish prognosis or direction of causation. These findings guide candidate selection and tissue-level testing; they do not test cross-scale association between human substitutions and fish phenotypes.

## Simple Summary

Scoliosisis a condition in which the spine develops an abnormal curve. Many genetic changes have been linked to scoliosis, but it remains difficult to determine which ones should be studied further and how to assess their effects on spinal tissues. We used computer models to examine previously reported human genetic changes and identified four candidates for further study. Two of these received more consistent support across the prediction methods. We also reanalysed published data from zebrafish with spinal curvature and found that the speed of waves travelling through their tissues was associated with the severity of the curve. Together, these analyses provide a framework for selecting genetic changes and choosing measurements for future experiments. The zebrafish studied here carried different genetic changes from the human candidates, so the findings do not establish that those candidates cause changes in spinal mechanics.

## 1. Introduction

Adolescent idiopathic scoliosis (AIS) emerges as a three-dimensional spinal deformity during growth, yet its biological origins remain difficult to assign [1,2]. Long-term outcomes vary, making curve progression and functional consequences distinct questions [3]. Bracing can reduce progression, demonstrating that the developing phenotype is modifiable without identifying its molecular origins [4]. Susceptibility loci near *LBX1*, within *GPR126* and at a *PAX1* enhancer implicate several developmental contexts [5,6,7]. Functional studies need both well-supported genetic candidates and measurements of the tissue phenotypes they may affect.

Common and rare variants provide complementary entry points into disease biology. Genetic evidence argues against treating AIS as a single molecular defect. Regulatory variation at *BNC2* and larger association studies implicate multiple loci, cartilage and connective-tissue development [8,9,10]. Rare-variant studies identify fibrillins and a distributed burden across musculoskeletal extracellular matrix (ECM) genes [11,12]. Exome studies also implicate variants in *FLNB* and *FNDC1*; their encoded proteins differ from secreted collagens [1,2]. The centriolar protein POC5 and manganese transporter SLC39A8 further broaden the relevant systems [13,14]. Association alone cannot specify a variant’s biochemical effect or tissue contribution. This heterogeneity makes gene-specific functional assessment necessary.

Folding stability captures one consequence of missense variation, but only part of protein function. Experimental stability maps quantify sequence effects, while structural predictions extend access to proteins with limited experimental coverage [15,16,17]. Structural coverage therefore expands the testable repertoire without resolving clinical interpretation. Model confidence and suitability require separate assessment [18]. Different structural signatures of loss-of-function, gain-of-function and dominant-negative mechanisms limit the use of destabilization as a uniform predictor of pathogenicity [19]. Filamin A shows force-sensitive partner binding, while skeletal-disease substitutions in human filamins can alter domain structure without eliminating every interaction [20,21]. Stability predictions can therefore help select substitutions for gene-specific assays, but cannot replace those assays.

Experimental assemblies can complement stability predictions by showing whether candidate sites are represented in resolved molecular complexes. Fibronectin fibrillogenesis depends on defined molecular regions and integrin translocation coupled to the actin cytoskeleton [22,23]. Experiments with fibronectin-deficient fibroblasts demonstrate that fibronectin and collagen-binding integrins help organize collagen deposition [24]. Cytoskeletal tension can extend and partially unfold assembled fibronectin, making interaction conformations dependent on loading [25,26]. Collagen assembly and fibronectin deformation also influence one another in cell-derived matrices [27,28]. Deposited complexes constrain where interactions occur, but do not describe assembly kinetics or the force-bearing state of living tissue.

Molecular attachments also regulate biochemical signalling under load. Latent transforming growth factor-β (TGF-β) is stored with its prodomain before release as an active signal. Its activation depends on molecular attachments that transmit force [29]. Experiments establish requirements for latent TGF-β binding protein 1 (LTBP1) in integrin-mediated activation and demonstrate release of active TGF-β1 by myofibroblast contraction [30,31]. Combined *Flnb* and *Ttc26* disruption in mice impairs Hedgehog signalling and intervertebral disc matrix homeostasis [32]. These findings motivate tissue-specific assays without assigning a single mechanism to every candidate allele.

Tissue mechanics depends on organization as well as constituent identity. Fibre networks can stiffen under strain; collagen connectivity and applied stress help determine their elastic response [33,34]. Cells reorganize fibres locally, altering their mechanical and adhesive environment [35]. Matrix elasticity influences lineage specification and regulators such as Yes-associated protein (YAP) and transcriptional coactivator with PDZ-binding motif (TAZ; PDZ refers to PSD-95/Dlg/ZO-1) [36,37]. Ligand anchoring and stress relaxation can also change cellular responses beyond a single elastic-modulus description [38,39]. Measurements of tissue organization must therefore be interpreted alongside their physical context. Maximum-to-minimum thickness variation describes one geometric feature, but does not measure connectivity, alignment or intrinsic stiffness.

Zebrafish models allow for tissue organization and mechanics to be examined during spine development. In zebrafish, the notochord sheath patterns vertebral regions, and notochord vacuoles accommodate compressive bone growth [40,41]. Disrupted cilia-driven cerebrospinal fluid (CSF) flow can produce scoliosis-like curvature [42]. The Reissner fibre and urotensin signalling further connect CSF-associated processes to spine development [43,44,45]. Pumputis and colleagues linked oxidative imbalance, matrix remodelling and elevated shear-wave velocity across zebrafish models [46]. Their measurements permit comparisons across distinct genetic backgrounds and treatment states. N-acetyl-L-cysteine ethyl ester (NACET) intervention provides a treatment-responsive setting for reanalysis.

The central question is how to prioritize scoliosis-related candidate substitutions and select quantitative tissue readouts for their subsequent functional assessment. The human analysis addresses which substitutions merit testing; the zebrafish reanalysis addresses what to measure and which controls to include. Within the tissue component, we evaluate the exploratory hypothesis that matrix thickness heterogeneity predicts treated-group mean squared shear-wave velocity more accurately than a constant untreated wild-type (WT) baseline. This baseline comparison was specified retrospectively. Interpreting shear-wave velocity requires assumptions about density, constitutive behaviour and geometry [47,48]. The fish perturbations involve *sspo*, *katnb1* and *vangl2*, whereas the human shortlist concerns *FLNB* and *FNDC1*. The analyses guide subsequent functional testing without assuming a shared mechanism across these perturbations.

## 2. Materials and Methods

### 2.1. Study Design and Source Units

This study integrates targeted computational analyses of public human genetic, structural and animal datasets. Human variants, structures and zebrafish measurements were analysed separately to support candidate selection and readout assessment; their perturbations and biological systems were not matched. It is not a new intervention experiment or a systematic meta-analysis; no laboratory reagents, devices, instruments, cell lines or physical samples were procured or handled. The animal data were extracted from the source workbooks accompanying Pumputis and colleagues [46]. Published mouse and *fndc1* zebrafish functional experiments inform the Discussion; their animal measurements were not included in our quantitative analyses. The source *sspo* model was the heterozygous $sspo^{dmh4/+}$ mutant. The extracted records comprised 209 shear-wave observations, 23 animal-level redox records, 140 ultrastructural segments and 20 paired shear-wave/Cobb-angle measurements. These counts refer to their respective analyses and were not summed to infer the number of unique animals across experiments. Age groups and source cohorts were analysed separately because cross-sheet reuse of animals could not be excluded. Source sheet and cell or row coordinates were retained. Constructed row identifiers were not treated as cross-sheet animal identities.

### 2.2. Variant Collection and Stability Calculations

Source variant observations were retained with study and phenotype labels, then collapsed by gene and protein substitution for variant-level calculations. Potentially overlapping genetic cohorts were not pooled. The purposive variant inventory used record-level lists from Haller et al., Jiang et al., Charng et al. and Yu et al. [1,2,12,49], selected for accessible substitution-level data in the musculoskeletal candidate genes considered here. It was not a systematic survey of all AIS genetic studies. Buchan et al. [11] informed the genetic background but supplied no directly extracted records in this inventory. Reference residues and isoform mappings were checked against UniProt protein sequences [50]; ambiguous mappings were excluded from the primary set. Nonsense substitutions were not scored as missense changes. AlphaFold Database models were used with their local confidence scores [16,17]. Predicted structures required a per-residue predicted Local Distance Difference Test (pLDDT) score of at least 70 for the primary comparison; experimental structures were handled separately from confidence scores.

ThermoMPNN predictions were generated using the pretrained model and released weights with unrepaired protein-only WT coordinates; EvoEF calculations used repaired wild-type structures and explicit mutant side-chain construction [51,52]. The main EvoEF reference was the globally repaired WT, following the published EvoEF workflow. Changes were reported as mutant minus wild type, with positive values denoting predicted destabilization. A separate sensitivity analysis used each mutant’s paired locally optimized WT, produced by the same neighbourhood-repacking routine. Both reference definitions and their candidate classifications are supplied. Fixed-backbone protein-only calculations omitted bound metals, glycans and explicit solvent molecules. Bound-metal and glycan effects were not modelled; EvoEF retained its implicit solvation terms. Predictions were not converted into clinical pathogenicity estimates. ClinVar provides submitted variant–condition interpretations and supporting assertions; these are distinct from computed folding effects [53]. Agreement above 1 kcal mol^−1^ in both methods defined an exploratory prioritization rule, rather than a physical transition or validated clinical cutoff. We also applied 0.5 and 1.5 kcal mol^−1^ cutoffs to the same 56 eligible substitutions. For a fixed cutoff, if the methods select *a* and *b* variants from *N*, random reassignment of one method’s labels gives expected overlap ab/N. The observed overlap was compared with the exact hypergeometric distribution, conditional on both marginal counts. A gene-stratified expectation was also calculated to assess gene composition. These tests quantify overlap under label exchangeability, rather than predictor accuracy or a biological null. For FBN1 N2178K, deposited nuclear magnetic resonance (NMR) models 1, 11 and 22 from Protein Data Bank (PDB) entry 1EMO were evaluated; for FBN1 N280T, models 1, 10 and 20 from PDB entry 5MS9 were evaluated. For these two substitutions, each variant-level estimate was the arithmetic mean of its three selected conformer predictions. The retained ranges describe these selected conformers, rather than full-ensemble uncertainty or confidence intervals. Variant-level resampling, rather than resampling NMR structures, was used for method-agreement summaries.

### 2.3. Targeted Third-Method Stability Assessment

The four shortlisted substitutions were evaluated using the DynaMut2 single-prediction application programming interface (API) [54]. Publicly available AlphaFold WT coordinates for FLNB and FNDC1 were submitted with chain A and sequence-verified substitution identities. Predictions were checked for chain, WT residue, position, mutant residue and a finite numerical value. Input 256-bit Secure Hash Algorithm (SHA-256) checksums, job identifiers, timestamps and unmodified responses were retained. DynaMut2 uses negative values for destabilization; we retain its raw predictions and multiply by −1 when presenting the manuscript’s positive-destabilization convention. This candidate-only follow-up did not rescreen the 56 variants, alter the original two-method rule or provide independent experimental validation.

### 2.4. Candidate Annotation and Human–Fish Sequence Correspondence

Genome-build-specific alleles were checked against transcript and protein consequences before querying the Genome Aggregation Database (gnomAD) r4 [55] and ClinVar (28 October 2026). Exome and genome allele counts and allele numbers were retained separately. Missing records and unreturned cohorts were not coded as zero frequency or benign status. ClinVar classifications were retained with condition, review status and accession version [53]. Archived per-protein AlphaMissense scores were reported separately from stability energies [56]; source Combined Annotation Dependent Depletion (CADD) values were checked against the original *FNDC1* workbook. For this revision, AlphaMissense annotations were completed across all 56 primary-eligible substitutions, including two *FBN1* records absent from the archived annotations. The released likely pathogenic cutoff of >0.564 was applied without optimizing it on the shortlist. We tested enrichment of this annotation in the four-variant shortlist using an upper-tail hypergeometric test conditional on the full-set count, with a gene-stratified calculation as a sensitivity check. These retrospective comparisons assess computational annotation concordance, not stability accuracy. Candidate alleles were additionally queried against GRCh38 CADD v1.7 and the University of California, Santa Cruz (UCSC) hg38 100-vertebrate phyloP track on 3 November 2026 [57,58]; phyloP was taken at the altered genomic nucleotide, rather than interpreted as an amino-acid or functional conservation measurement. Source-study CADD values were retained separately from current-version scores. Human UniProt FLNB O75369 and FNDC1 Q4ZHG4 were globally aligned to retrieved zebrafish *flnba*, *flnbb* and *fndc1* sequences using BLOSUM62 with gap-open/extension scores of −10/−0.5 and −12/−1. Specific accessions, isoforms, residue correspondences and local 31-residue identities are supplied; sequence similarity does not establish conserved function, tissue expression or disease mechanism.

### 2.5. Experimental Biological Assemblies and Interface Mapping

Biological assembly 1 was analysed for each of 70 selected PDB entries [59] covering a selected panel of 35 proteins. Deposited assembly annotations were used rather than assuming that an asymmetric unit represented the biological oligomer. The complete protein panel, accessions, entry-selection records and entry-specific provenance are supplied in Supplementary Note S6 and Supplementary Data S2 and S3. This was a purposive structural-context panel, not an exhaustive search or an unbiased interaction-network sample. Twelve genetics-motivated proteins were supplemented by 23 proteins spanning matrix components, receptors, remodelling, adhesion and mechanosensing/signalling, to provide deposited structural context. Research Collaboratory for Structural Bioinformatics (RCSB) queries used exact UniProt accessions and required at least two protein entities or at least two polymer instances, with fewer than 180,000 deposited atoms. Up to four entries per panel gene were retained, prioritizing broad interactions through at least three protein entities, then electron-microscopy method, better resolution and entry identifier in the archived order. The union comprised 70 entries; proteins without a selected entry remained in the 35-protein panel. Structure Integration with Function, Taxonomy and Sequences (SIFTS) mappings linked deposited residues to UniProt positions [60]. Experimental coordinates remained separate from AlphaFold predictions used in the candidate stability screen.

The first deposited model was used. Heavy atoms with alternative-location identifiers absent or A were retained after duplicate atom removal. Protein atoms included hydroxyproline and selenomethionine where present; non-protein glycans and ions were excluded. Candidate chain pairs required at least three contacting residues on each partner within 5 Å. Primary interfaces additionally required one-sided buried surface area of at least 100 Å^2^. Shrake–Rupley surfaces were calculated with MDTraj, using a 1.4 Å probe and 240 sphere points per atom [61,62]. One-sided buried area was half the summed non-negative per-atom surface loss upon pairing the chains. This is a coordinate-derived surface measure, not an energetic calculation.

Pairs with mapped panel labels on both partners were collapsed to unordered protein pairs only when each chain mapped to a single UniProt accession. Twelve chain pairs from four type-I/type-IX collagen chimeric constructs (5CTD, 5CTI, 5CVA and 5CVB) were excluded from protein-specific area aggregation; their raw contact measurements were retained as construct context. This avoids assigning a whole chimeric chain’s buried surface to one component protein. Domain and peptide constructs remained eligible, with their molecular scope stated explicitly. Reported means and minima/maxima describe the retained chain-pair areas; assembly counts are the numbers of distinct PDB entries. Multiple chain pairs in an entry were not treated as independent experimental replicates, and no confidence intervals were inferred from these descriptive ranges. Exact variant mapping required concordant reference amino acids and canonical sequence positions; missing coverage was distinguished from covered sites outside retained interfaces.

Numerical sensitivity compared 240 with 960 sphere points for ten chain pairs from three representative entries. Threshold tabulations applied area cutoffs of 50, 100, 200 and 500 Å^2^ and at least three atom-pair contacts within 4, 5 or 6 Å to the initially selected, panel-mapped chain pairs. The initial 5 Å contacting-residue selection remained fixed; these tabulations were not independent rescreenings of all possible interfaces. Assembly results did not enter stability prioritization, geometry calibration or curvature prediction.

### 2.6. Mechanical Effects and Intervention Analysis

For animal *i*, the mechanical proxy was $g_{i}\;=\;v_{i}^{2}$. Group means were calculated as $\bar{v^{2}}$, not ${\left(\bar{v}\right)}^{2}$. For a homogeneous, isotropic, linear elastic bulk medium, shear-wave speed satisfies $v_{s}\;=\;\sqrt{\mu /\rho }$. Under equal density, ratios of mean squared speeds therefore provide a dimensionless comparison of this simplified modulus proxy [47]. Conversion to Young’s modulus additionally requires Poisson’s ratio, through E = 2μ(1 + ν); we do not perform this conversion. The source tissue is layered, and anisotropy, prestress, viscosity, frequency and boundary geometry can alter wave propagation [48]. Neither equal density nor bulk-wave behaviour was verified here. Accordingly, $g_{i}$ is reported in m^2^s^−2^ as a mechanical readout, without assigning an intrinsic modulus in pascals. Within each source comparison, mutant-to-control ratios were calculated from group means. WT denotes the reference-group code in the processed tables; in both *katnb1* experiments, reference animals are *katnb1*/+ heterozygotes rather than wild type. Animals were resampled independently within groups for 10,000 bootstrap replicates using seed 260917. Within-source group comparisons used two-sided Welch’s *t*-tests on log-transformed proxy values and the Benjamini–Hochberg correction within the ten-comparison family, which included treatment contrasts as well as the six mutant–control comparisons. For the *sspo* mutant-minus-WT velocity difference, a separate 10,000-replicate animal bootstrap reset seed 260917 before resampling. The *sspo* intervention was analysed with a linear model containing genotype, treatment and their interaction; the reported interaction is on the velocity scale. Technical regions of interest were not treated as independent biological samples. Residual-versus-fitted, normal quantile–quantile and Cook’s-distance plots were inspected for the velocity interaction and Cobb models. The Shapiro–Wilk and studentized Breusch–Pagan tests were descriptive checks; heteroscedasticity-consistent HC3 covariance estimates provided sensitivity intervals using residual-df *t* critical values. No observation was excluded on the basis of these checks (Supplementary Note S5; Figure S2). For illustrative sample-size planning, we used the pooled within-group shear-wave velocity variance from the untreated 10-day source groups (15 WT and 15 *sspo* animals; 0.320 m^2^s^−2^). Equal-allocation, two-sided two-sample *t*-test calculations used α = 0.05 and 80% power. Differences of 0.25 and 0.50 m s^−1^ were planning scenarios, rather than estimated effects of the candidate missense alleles; these calculations did not include attrition or clustering.

### 2.7. Untreated-to-Treated Calibration

Let $G_{k}$ denote the mean squared velocity in group *k*, and $Q_{k}$ its mean within-segment maximum-to-minimum thickness ratio. Geometry calibration used

(1) $${\hat{G}}_{k}=G_{\mathrm{WT}}{\left(\frac{Q_{k}}{Q_{\mathrm{WT}}}\right)}^{\gamma },\;\gamma =\frac{\log(G_{\mathrm{Mut}}/G_{\mathrm{WT}})}{\log(Q_{\mathrm{Mut}}/Q_{\mathrm{WT}})}.$$

Only untreated groups were used to determine γ = 3.593 and the scale. Both NACET groups were withheld. The relative error was $({\hat{G}}_{k}/G_{k}\;-\;1)\;\times \;100\%$. The constant-control comparator predicted ${\hat{G}}_{k}\;=\;G_{\mathrm{WT}}$ for each withheld group, without using treated-group outcomes. We compared relative errors and root-mean-square error (RMSE) for the two models over the same two withheld means; this baseline was specified retrospectively. This is a group-level calibration, with no training residual degrees of freedom. The geometry table contained 36, 47, 22 and 35 segments in untreated WT, untreated mutant, treated WT and treated mutant groups, respectively. Published animal totals were 8, 9, 6 and 7, but segment identities could not be assigned back to animals. We retained segment-weighted means and did not calculate pseudoreplicated animal-level intervals.

The redox model replaced $Q_{k}$ with the mean background-corrected fluorescence $R_{k}$. Three regions of interest were first averaged within each animal. Redox group sizes were 3, 8, 3 and 9; mechanical group sizes were 15 each. Independent within-group animal bootstraps propagated uncertainty from the separate redox and mechanical cohorts. Treatment values did not enter exponent fitting. This design estimates a relationship between group means and cannot identify within-animal mediation or individual prediction intervals.

### 2.8. Cobb-Angle Analysis

The 20 paired observations were extracted from panel (e) of the source study’s Cobb-angle/velocity plot [46] and comprise 21-day-post-fertilization *sspo* mutants measured after curve onset. The workbook column is labelled Most Severe Cobb Angle; the source figure legend defines this as the most severe angle in the sagittal or coronal plane. The supplied scalar was retained without recomputing or summing angles across planes. A linear regression of this angle on shear-wave velocity was fitted to the 20 paired observations. Coefficient intervals were calculated from the fitted model. For leave-one-animal-out validation, the regression was refitted after removing each animal, and the withheld angle was predicted once. The baseline prediction was the mean angle of those same training animals. We additionally drew 20,000 case-bootstrap samples (seed 261028), refitting both models in each sample. All repeated copies of a held-out original animal were excluded together, and remaining bootstrap multiplicities were used as regression weights. This avoids identity leakage between training and test sets. Percentile ranges describe internal resampling uncertainty, without treating overlapping folds as independent replicates. Resamples contained a median of 13 distinct animals, so these intervals do not establish nominal population coverage or replace external validation. Root-mean-square errors were computed over the 20 withheld predictions. This refitting design estimates prediction error without testing each animal on a model that included its own outcome [63]. The alternative log-proxy model is retained with the distributed processed results for internal comparison; it was not combined with the velocity model to create an optimized predictor.

### 2.9. Physical Diagrams and Molecular Structure Figures

Geometry and wave-propagation diagrams are explanatory drawings with no spatial or temporal calibration. Paired segment thicknesses are the original source measurements, and the geometry-to-mechanics calibration is presented in the Results.

Molecular views use AlphaFold wild-type coordinates and the paired locally optimized wild-type and mutant coordinate files from the corresponding EvoEF1 calculations [16]. Each prioritized position was verified against wild-type and mutant residue identities. Cartoon representations show local wild-type sequence windows of up to 60 residues on each side, truncated at the protein terminus; site confidence is the AlphaFold pLDDT value. Right-handed principal-axis orientations were calculated in R version 4.4.2. Where the nominated residue was obscured, local-fold views were rotated to improve its visibility without changing the coordinates or residue window. The paired WT views correspond to the alternative-reference models in the stability sensitivity analysis; local fold cartoons use the original AlphaFold WT coordinates. Each wild-type/mutant pair uses the same wild-type-defined orthographic camera and coordinate scale. Up to three neighbouring residues were selected by their nearest heavy-atom distance to the wild-type substitution site, with a 4 Å cutoff, and retained for both models. Molecular structures were rendered in Mol* 5.11.0 as cartoons and ball-and-stick models, with thin sticks for neighbouring residues, without changing the input coordinates. Structural plates and labels were assembled and the final images were exported using R. A common crop and display size were applied to each wild-type/mutant image pair, preserving their relative coordinate scale.

### 2.10. Data, Code and Reporting

The computational software versions were Python 3.15.2, ThermoMPNN v1.0.0, EvoEF1, DynaMut2 and MDTraj v1.11.2; R 4.4.2 and Mol* 5.11.0 are specified above. CADD v1.7 was used for variant annotation. R was used for quantitative figures; Python was used for data processing. Input checks verified genotype labels, background correction, thickness ratios, sequence mappings, structural eligibility, WT-reference energies and panel membership. Supplementary Data S1–S4 provide the complete inventories and processed results. Historical full ThermoMPNN/EvoEF inference, atom-level surface recalculation and native Mol* rendering were not repeated in the numerical replay. The targeted DynaMut2 predictions are documented with their raw responses. The accompanying revision supplement (v2) also includes the historical scoring structures for all 56 eligible substitutions: 60 model–variant records, including three conformers for each *FBN1* substitution, with 138 coordinate files covering original WT, globally repaired WT, paired locally optimized WT and mutant models. File hashes, target-residue identities, model mappings and the eight original candidate-coordinate hashes were checked. The numerical replay and revision-specific checks were executed successfully; full stability inference was not rerun. Original project code and documentation are licensed under MIT, with third-party material retaining its own terms. The versioned supplement (v2) is archived at https://doi.org/10.5281/zenodo.23906016. The code and data are publicly available at https://github.com/azusakou/scoliosis-variant-and-zebrafish (accessed on 7 September 2026). No new human or animal measurements were collected.

## 3. Results

### 3.1. Stability Predictions Prioritize Four Human Substitutions

We assembled variant records from published human genetic studies [1,2,12,49], retaining each study’s phenotype and source labels. The atlas contained 121 deduplicated gene–substitution records. Nineteen were not scored: ten had ambiguous isoform mappings, one had a reference-residue mismatch, two were non-missense consequences and six *FBN1* substitutions lacked sequence-matched experimental coordinates. Of 102 scored substitutions, 12 tentative isoform mappings and 34 sites with pLDDT below 70 were excluded from the primary comparison. The remaining 56 comprised two *COL11A2*, two *FBN1*, 17 *FLNB* and 35 *FNDC1* substitutions. Supplementary Note S4 and Supplementary Data S1 give the complete records and inclusion decisions. Source populations were not pooled for a new case–control analysis, and AIS case, control-enriched and other phenotype labels were retained. Atlas inclusion consequently did not classify every record as an AIS risk allele.

**Figure 1:**
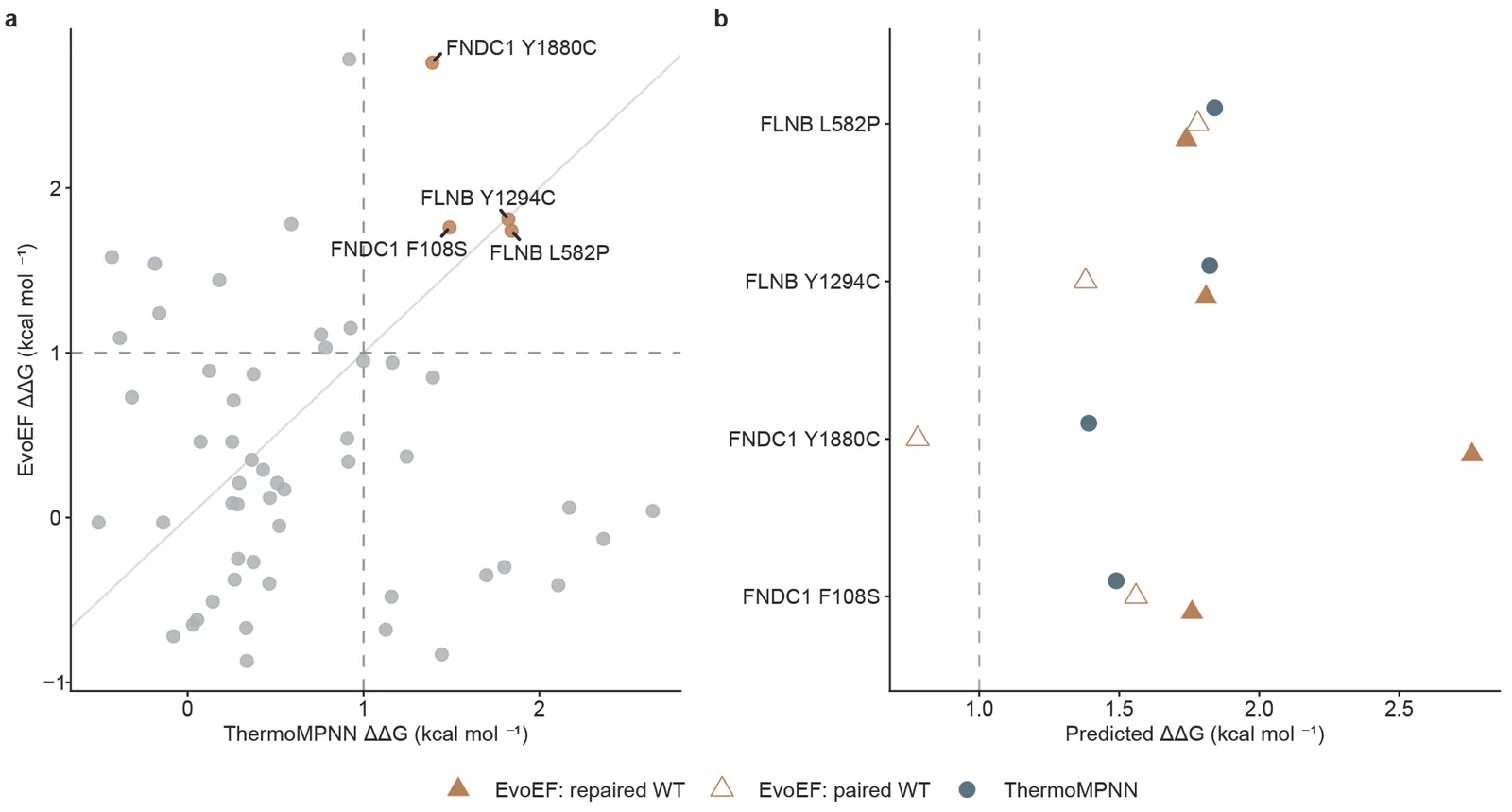
Exploratory stability shortlist. The four candidates are *FLNB* L582P/Y1294C and *FNDC1* F108S/Y1880C. (**a**) Predictions for 56 eligible substitutions; orange points exceed 1 kcal mol^−1^ in both methods (dashed lines). EvoEF uses globally repaired wild type (WT). Four overlaps were observed and four expected by chance. (**b**) The four candidates; open symbols show the alternative paired-WT EvoEF estimates. Y1880C falls below threshold under this reference. These predictions lack experimental validation.

ThermoMPNN and EvoEF showed weak agreement across the primary set (Spearman’s correlation, 0.038). Four substitutions exceeded the exploratory 1 kcal mol^−1^ threshold in both methods: *FLNB* L582P and Y1294C, and *FNDC1* F108S and Y1880C (Figure 1; Table 1). ThermoMPNN selected 16 and EvoEF 14 of 56 substitutions, giving an independent-label expected overlap of 16 × 14/56 = 4.0. The observed overlap of four did not exceed this expectation (exact upper-tail hypergeometric p = 0.624; gene-stratified expected overlap, 4.191).

At cutoffs of 0.5 and 1.5 kcal mol^−1^, observed joint counts were 12 versus 10.125 expected (p = 0.224) and two versus 1.143 expected (p = 0.320), respectively; the latter substitutions were *FLNB* L582P and Y1294C. Across all three cutoffs, the two-method rule showed no evidence of overlap beyond chance conditional on the individual selection counts, and therefore supplied no demonstrated additional selection signal over the separate scores. Using the paired locally optimized WT as the alternative EvoEF reference retained three candidates above both thresholds: *FLNB* L582P and Y1294C, and *FNDC1* F108S. *FNDC1* Y1880C fell to 0.78 kcal mol^−1^ and is therefore reference-sensitive (Supplementary Note S3). Table 1 reports all estimates under both reference definitions. The resulting shortlist is exploratory and depends on the threshold and WT reference.

DynaMut2 returned positive-destabilization estimates of 0.219, 1.557, 2.090 and −0.301 kcal mol^−1^ for L582P, Y1294C, F108S and Y1880C, respectively. Only Y1294C and F108S exceeded 1 kcal mol^−1^ in this four-candidate follow-up. L582P had a smaller predicted effect, whereas Y1880C had the opposite direction (Table 1; Supplementary Note S4). These differences distinguish computational support within the shortlist without providing experimental validation or a three-method ranking of all 56 variants. The full-set AlphaMissense analysis classified 19 of 56 substitutions as likely pathogenic, including all four shortlisted variants (conditional expected overlap, 1.357; exploratory upper-tail p = 0.0106). Thus, the shortlist was enriched for this computational pathogenicity annotation, although this does not establish thermodynamic agreement, clinical accuracy or benefit over either stability tool alone. The full 56-row table and gene-stratified sensitivity calculation are supplied in the code/data supplement.

Table 1 separates the four candidates’ scores, coverage and first tests. ClinVar searches on 28 October 2026 retrieved uncertain-significance records for *FLNB* Y1294C and *FNDC1* F108S/Y1880C, each from a single submitter; no matching L582P record was retrieved. The gnomAD r4 exome frequencies were $3.42\;\times \;10^{-6}$, $8.94\;\times \;10^{-6}$ and $2.10\;\times \;10^{-4}$ for these three alleles. L582P returned no matching variant, rather than a measured zero frequency. AlphaMissense scores ranged from 0.7916 to 0.9715; these are computational pathogenicity predictions, not functional or clinical validation [56]. Precise alleles, accession versions, cohort-specific counts and source CADD values are provided in Supplementary Note S4 and Supplementary Data S1. Table 1 adds the population frequencies, ClinVar status, uniform CADD v1.7 scores and multi-species nucleotide conservation. *FNDC1* Y1880C had a non-Finnish European exome frequency of $2.28\;\times \;10^{-4}$ (253/1,111,348 alleles; one homozygote), relevant to the European-ancestry source cohort [2]. Frequencies for all returned ancestry groups and separate genome counts are provided in Supplementary Note S4 and the code/data supplement. This frequency and the observed homozygote do not support interpreting Y1880C as a highly penetrant cause of severe AIS; a lower-penetrance contribution remains untested for this individual allele.

To assess cross-species correspondence, we compared the existing human–fish alignments by zebrafish accession under both gap settings. *FLNB* L582 and Y1294 aligned to L582 and Y1292 in both retrieved *flnba* entries and to L393 and Y864 in three of four *flnbb* entries. In those three *flnbb* entries, the local windows contained 20 identical residues among 31 aligned positions around L582 and 11 among 29 around Y1294. Neither site mapped in the shorter fourth *flnbb* entry. *FNDC1* F108 aligned to F132 in *fndc1* A0AB40E856 but had no aligned position in the other retrieved entry; human Y1880 aligned to phenylalanine in both. These comparisons identify sequence-compatible sites and accession-dependent gaps, rather than establish conserved function or effects in the measured fish (Supplementary Data S1).

**Figure 2:**
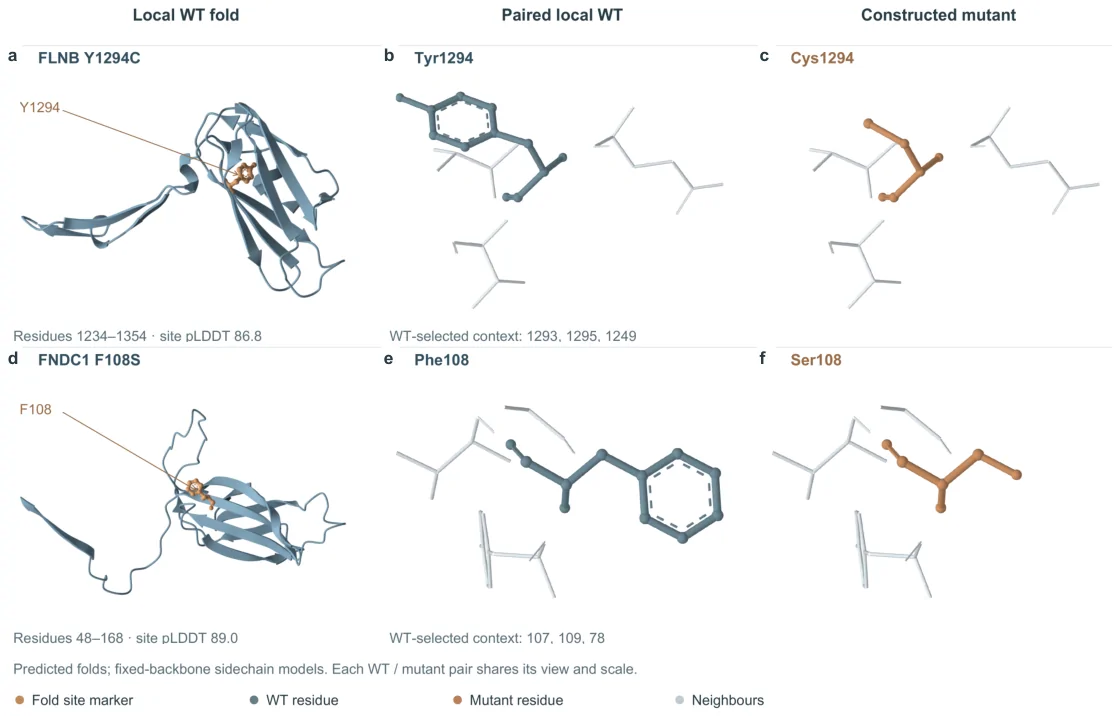
Structural localization of two candidates with consistent threshold classification across three methods. Rows: *FLNB* Y1294C (**a**–**c**) and *FNDC1* F108S (**d**–**f**). **Left**: Local AlphaFold wild-type (WT) folds and labelled residues, with predicted Local Distance Difference Test (pLDDT) site scores of 86.8 and 89.0. **Middle**/**right**: Paired locally optimized WT/EvoEF-built mutant side chains, sharing camera and scale. Neighbours were selected within 4 Å in WT. These constructions locate substitutions; they do not establish changed packing or function. Corresponding L582P and Y1880C views appear in Supplementary Figure S3; all four candidates remain in Table 1.

Coordinate views localize *FLNB* Y1294C and *FNDC1* F108S, the two candidates exceeding the threshold in all three methods (Figure 2). Their site pLDDT values were 86.8 and 89.0. The corresponding views for method-discordant L582P and reference- and method-sensitive Y1880C are retained in Supplementary Figure S3. Table 1 retains all four candidates and their scores. Paired locally optimized WT and fixed-backbone mutant side chains provide structural localization, without establishing experimental mutant conformations or functional effects.

### 3.2. Experimental Assemblies Delimit Structural Coverage

The 70 selected assembly-1 structures yielded 54 panel-labelled chain pairs after contact, buried-area and single-accession filtering. These represented 14 protein pairs, including six heterotypic contacts involving collagen, FN1, integrins and LTBP1–TGFB1. The complete contact analysis, construct-specific interpretation and numerical sensitivities are provided in Supplementary Note S1 and Figure S1. Only *FLNB* M1803L and R199Q had exact source-variant coverage, and neither lay at a retained interface. None of the four nominated substitutions was covered. This coverage audit therefore documents a gap in experimental structural evidence; it supplies no interface mechanism for these candidates and was not used in the tissue models.

### 3.3. Shear-Wave Responses Across Zebrafish Models

At the tissue scale, we assessed whether a common mechanical readout could be compared across published genetic perturbations. The published source data comprised 209 shear-wave observations across *sspo*, *katnb1* and *vangl2* experiments. The two *katnb1* experiments used *katnb1*/+ heterozygotes as controls, rather than wild type. We analysed each genotype and age against its corresponding control, without pooling potentially reused animals across source sheets. Squared shear-wave velocity, $v^{2}$, served as a modulus proxy under constant-density and simplified wave-propagation assumptions; it was not treated as a measured Young’s modulus.

**Figure 3:**
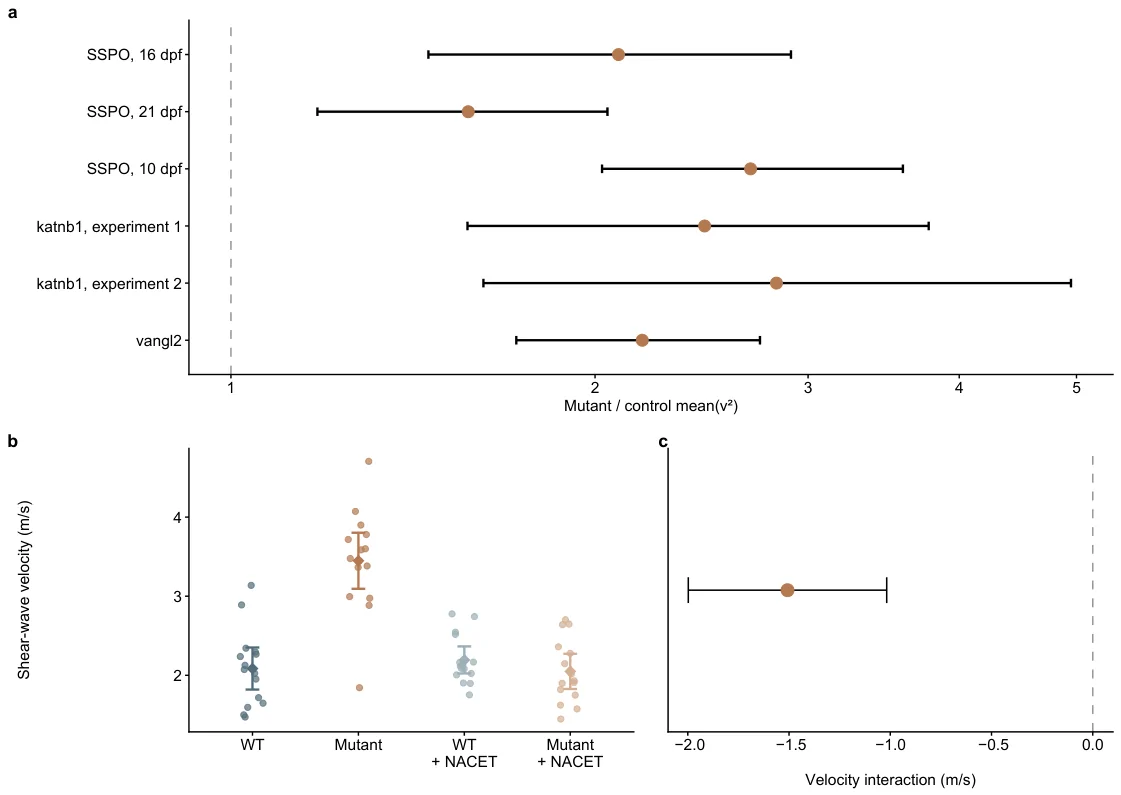
Shear-wave responses across zebrafish models. Data are from zebrafish *sspo*, *katnb1* and *vangl2*; WT denotes wild type. (**a**) Mutant/control mean ($v^{2}$) ratios; percentile 95% intervals from 10,000 within-group animal bootstraps. Mutant/control sizes, top to bottom: 15/15, 15/15, 15/15, 11/11, 9/9 and 15/15. Both *katnb1* controls are heterozygotes; comparisons belong to a ten-test Benjamini–Hochberg family. (**b**) Ten-day *sspo* velocities: Animals (n = 15/group), means (diamonds) and 95% *t* intervals. (**c**) Genotype-by-N-acetyl-L-cysteine ethyl ester (NACET) coefficient with ordinary 95% interval (−1.51 m s^−1^, $p\;=\;8.18\;\times \;10^{-8}$); the jackknife heteroscedasticity-consistent covariance estimator (HC3) sensitivity appears in Supplementary Note S5. Dotted vertical reference lines indicate a ratio of 1 in panel (**a**) (no mutant/control difference) and zero interaction in panel (**c**).

All six mutant–control comparisons showed increases in this proxy (Figure 3a). At 10 days post-fertilization, the *sspo* mutant-to-control ratio was 2.69 (bootstrap 95% confidence interval, 2.03–3.59); the corresponding ratios at 16 and 21 days were 2.09 and 1.57. The two *katnb1* comparisons yielded ratios of 2.46 and 2.82, and the *vangl2* comparison yielded 2.19. All six comparisons retained support after correction within the specified test family. These estimates quantify a concordant response across the analysed experiments, rather than a longitudinal trajectory or a universal property of scoliosis-associated variants.

The *sspo* intervention data allowed for the wave response to be assessed under treatment (Figure 3b,c). At 10 days, mutant shear-wave velocity exceeded that of the untreated wild type by 1.36 m s^−1^ (95% confidence interval, 0.97–1.75). The genotype-by-NACET interaction was −1.51 m s^−1^ (95% confidence interval, −2.00 to −1.02; $p\;=\;8.18\;\times \;10^{-8}$), with treated mutant means approaching untreated wild-type values. These patterns reproduce the published intervention response [46]; the reanalysis quantifies their effect sizes and uncertainty. Residual diagnostics showed tail deviations in the velocity model (Shapiro–Wilk test, p = 0.0506), while the HC3 interaction interval remained −2.015 to −1.000 m s^−1^ (Supplementary Note S5).

### 3.4. Untreated-to-Treated Transfer of Geometry-Based Calibration

**Figure 4:**
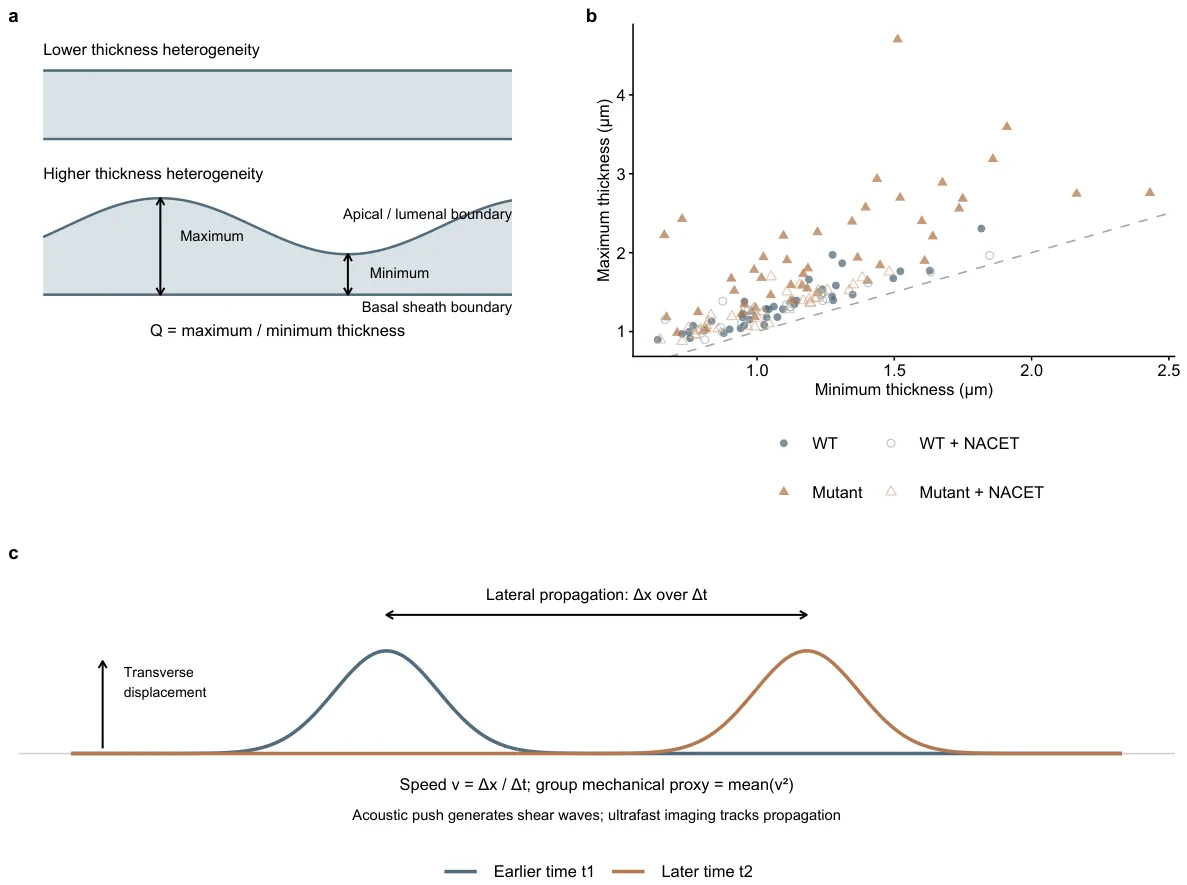
Tissue thickness and shear-wave measurement. (**a**) *Q* is maximum/minimum thickness measured normal to the basal boundary. (**b**) All 140 paired segments: 36 wild type (WT), 47 mutant, 22 WT+N-acetyl-L-cysteine ethyl ester (NACET) and 35 mutant+NACET. Dashed line, equal thicknesses. Missing animal assignments prevent treating segments as independent animals. (**c**) Shear-displacement schematic defining v = Δx/Δt. Schematics (**a**,**c**) lack spatial/temporal calibration. Mean ($v^{2}$) is a mechanical proxy under unverified density, constitutive and propagation assumptions.

We then tested whether matrix organization carried information about the treated mechanical state. The ultrastructural dataset contained 140 segments with paired maximum and minimum thickness measurements. Mean within-segment thickness ratios were 1.23 in untreated wild type and 1.62 in untreated mutants, falling to 1.25 in treated mutants. The source sheet did not retain animal identifiers for individual segments. We therefore used segment-weighted group means and did not interpret the segment count as the number of independent animals. Figure 4 illustrates the geometry definition, all observed thickness pairs and the measurement-to-model relationship.

**Figure 5:**
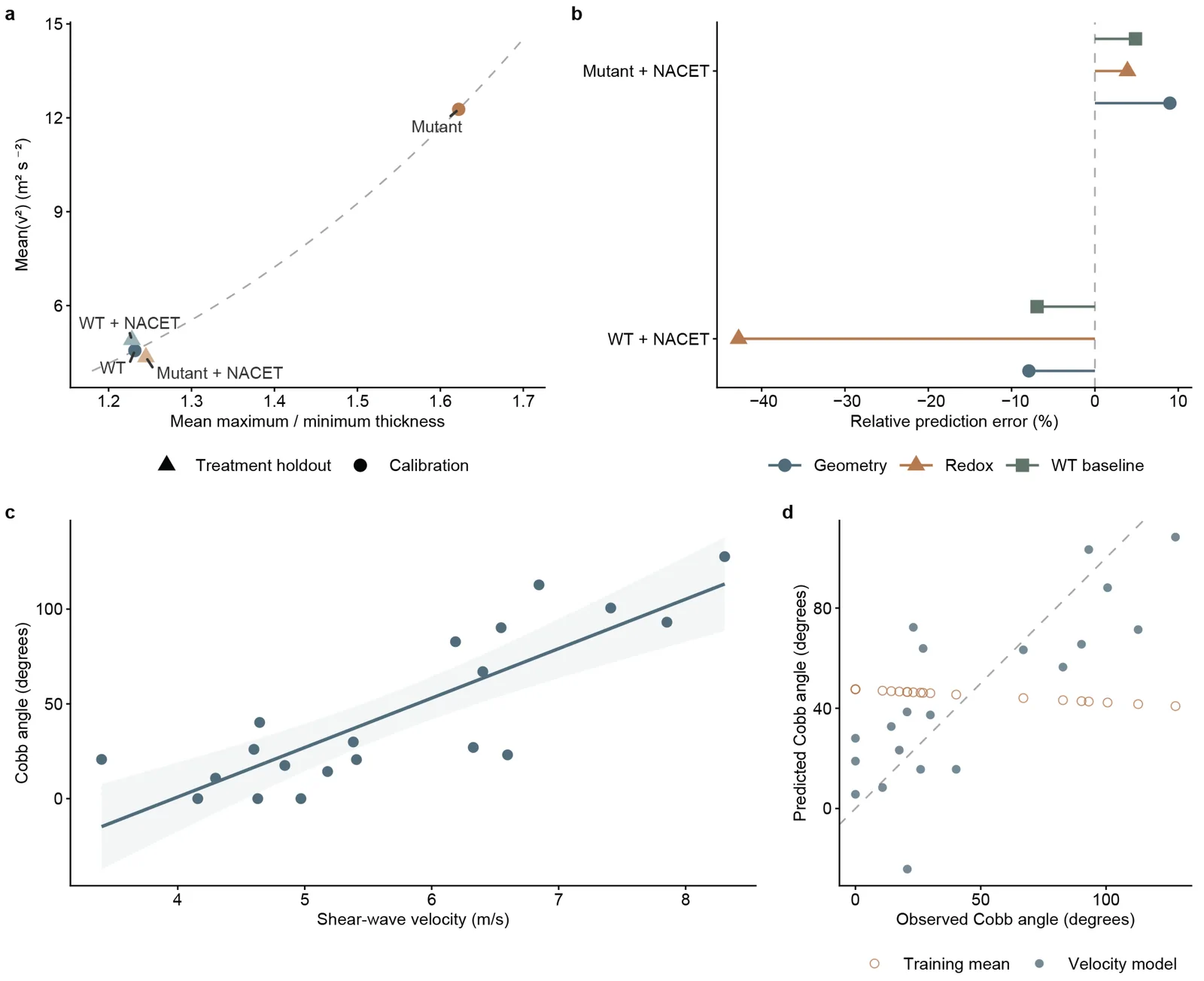
Calibration transfer and internal curvature prediction. (**a**) Geometry and mean ($v^{2}$): Two untreated calibration means (circles) and two withheld treatment means (triangles). Sampling is specified in Figure 3 and Figure 4 and Supplementary Note S2. (**b**) Geometry, redox and constant-wild-type (WT) relative errors; lines are not uncertainty intervals. The constant-WT baseline outperforms geometry in both groups. (**c**) Twenty post-onset *sspo* velocity/angle pairs, linear fit and 95% conditional-mean band ($R^{2}\;=\;0.698$); angles mix sagittal/coronal planes. (**d**) Leave-one-animal-out predictions versus observed angles for the velocity model and training-mean baseline; dashed line, identity. Negative predictions are retained. Their respective root-mean-square errors (RMSEs) are 24.6°/42.3°, with 95% internal resampling intervals 17.5°–35.0°/30.7°–53.9°.

A power-law description calibrated on two untreated group means (Figure 5a) was evaluated against the constant untreated-WT baseline in two withheld treatment groups (Figure 5b; Supplementary Note S5). For treated WT and mutants, geometry predicted 4.52 and 4.74 m^2^s^−2^ versus observed means of 4.90 and 4.35, giving errors of −7.9% and +9.0%. The constant baseline predicted 4.564 m^2^s^−2^ for both groups, with smaller errors of −6.95% and +4.87%. Across the two means, RMSE was 0.390 for geometry versus 0.284 m^2^s^−2^ for the baseline. This comparison did not support additional predictive information from thickness heterogeneity.

No treatment-group outcome entered calibration. Two untreated means determine both parameters, leaving no training residual degrees of freedom. Missing segment-to-animal assignments prevent reliable animal-level uncertainty estimates, and unequal segment contributions may affect group means. The fitted relation therefore remains a limited group-level calibration rather than a validated thickness-based predictor.

Redox measurements supplied another exploratory group-level comparison. Averaging three regions within each of 23 animals gave an untreated-group exponent of 0.545 (bootstrap 95% interval, 0.262–1.299). Errors were +3.9% in treated mutants and −42.8% in treated WT. With only three, eight, three and nine animals per redox group, and separate mechanical cohorts, these two errors cannot establish general model superiority or individual mediation.

### 3.5. Mechanical Measurements Carry Information About Spinal Shape

To examine mechanical information at the individual-animal scale, we analysed 20 *sspo* mutants at 21 days post-fertilization with paired shear-wave and source-reported most-severe Cobb-angle measurements, acquired after curve onset. The association was reported in the source study; here we quantified its fitted effect and internal predictive error [46]. A linear model explained 69.8% of the observed angle variation (Figure 5c). The fitted slope was 26.1° per m s^−1^ (95% confidence interval, 17.6°–34.6°). The HC3 sensitivity interval was 16.83°–35.36° per m s^−1^ (Supplementary Note S5). Residual checks gave W = 0.980 (p = 0.933) for the Shapiro–Wilk test and p = 0.708 for the studentized Breusch–Pagan test. The largest Cook’s distance was 0.389; one of 20 observations exceeded the descriptive 4/n = 0.2 reference, and no animal was excluded.

Leave-one-animal-out refitting gave a root-mean-square prediction error of 24.6°, compared with 42.3° for the training-mean baseline (Figure 5d). Animal-level case resampling excluded all copies of each held-out identity during weighted refitting. The resulting 95% internal resampling intervals were 17.5°–35.0° for regression RMSE and 30.7°–53.9° for the baseline. These intervals quantify finite-sample instability, not external accuracy or individual prediction limits (Supplementary Note S5). The model retained information beyond the sample mean but still had substantial error. The source outcome selects the larger sagittal or coronal angle, and measurements were acquired after curve onset. The association therefore cannot distinguish mechanics preceding curvature from mechanics altered by existing curvature. The separate group-level geometry analysis does not establish individual geometry-to-curvature prediction or temporal mediation.

## 4. Discussion

Within the four-candidate follow-up, *FLNB* Y1294C and *FNDC1* F108S showed the most consistent threshold classification across methods. L582P and Y1880C remained method-dependent, with Y1880C also sensitive to the WT reference. In the fish data, shear-wave velocity varied with genotype and treatment, whereas thickness heterogeneity did not improve prediction in the two treated groups. The results distinguish computational support within the molecular shortlist and clarify the controls needed to interpret tissue measurements in subsequent functional studies.

FLNB substitutions can alter folding while retaining ligand binding [21]; the source study’s interaction assays for R566L, A2282T and R2003H do not validate L582P or Y1294C [1]. Two functionally studied substitutions, R566L and R2003H, were present in the 56-variant primary set but were not prioritized. Their ThermoMPNN/EvoEF estimates were 0.46/−0.40 and 0.59/1.78 kcal mol^−1^, respectively. The published interaction assays, including variant-partner combinations, did not measure folding stability and are not a strict stability benchmark. Nevertheless, these omissions illustrate that a stability-only intersection can miss substitutions relevant to interaction phenotypes. Partner binding, localization and force-dependent regulation therefore require separate gene-specific tests. Published models point to different spinal tissue contexts for these genes. Combined *Flnb*/*Ttc26* disruption impaired Hedgehog–GLI2 signalling and disc matrix maintenance in mice. Joint knockdown in wild-type tail discs reduced compression moduli [32], a result attributable to the combined intervention. Zebrafish carrying an *fndc1* nonsense allele showed increased vertebral bone mineral density and cross-sectional area, without increased scoliosis reported [2]. That study assessed bone phenotypes without direct mechanical tests. These findings guide gene-specific tissue and assay selection without requiring a common ECM mechanism.

None of the shortlisted sites was covered by the surveyed experimental assemblies. The fibronectin, collagen and integrin complexes represent distinct recognition contexts [64,65,66,67,68], while LTBP1–latent TGF-β provides another resolved interaction [29]. Missing site coverage limits structural interpretation of the candidates but does not exclude an interface effect. Buried surface areas describe deposited conformations and cannot establish affinity, force transmission or physiological importance. The detailed contact analysis is retained in the Supplementary Information.

Pumputis and colleagues established the genotype effects, NACET response and velocity–curvature association [46]. Using their observations, we assessed calibration transfer, introduced a constant-WT comparator and quantified uncertainty and model diagnostics. With two fitted parameters, the geometry calibration reproduces both untreated means exactly. The two treated means provide a separate, small within-study test, but their thickness ratios lie close to the WT calibration point. The constant-WT baseline had smaller errors in both groups, so this comparison does not support added predictive value from geometry. The fitted exponent, 3.593, describes the sampled range and is not a material constant.

Collagen connectivity and applied stress influence collective mechanics [33,34], but the maximum-to-minimum thickness ratio measures neither connectivity nor fibre orientation. It also discards absolute thickness, because uniformly rescaling a profile leaves the ratio unchanged. Thickness heterogeneity therefore needs to be interpreted alongside absolute geometry, composition and alignment. Experiments separating stress relaxation or ligand anchoring from initial elasticity illustrate why these material features require distinct measurements [38,39].

Thickness can affect wave propagation as well as tissue organization, complicating interpretation of the geometry calibration [48]. Squared shear-wave velocity reflects a shear modulus only under the bulk-medium and constitutive assumptions described in the Methods. The present data cannot separate material changes from geometric propagation effects. Frequency-dependent wave measurements and independent local mechanical tests would help distinguish them; the current calibration target remains mean squared velocity.

Oxidative stress can affect matrix turnover through cell signalling and matrix chemistry [26]. In cartilage models, inflammatory responses to reactive oxygen species, including NF-κB signalling, can accompany senescent or hypertrophic programmes, increased matrix-degrading enzymes and reduced matrix synthesis [69]. These pathways offer a possible explanation for redox-associated tissue changes, but were not assayed here. In the source fish experiments, NACET reduced abnormalities in collagen orientation and matrix thickness [46]. Fluorescence samples a biochemical reporter at a particular time, whereas structure may integrate earlier remodelling. The separate cohorts, small and unequal redox groups and two treated means leave this temporal explanation untested. Uneven redox transfer therefore does not identify geometry as the better predictor or contradict the published mechanism. Testing the explanation would require biochemical, structural and mechanical measurements in the same animals over time.

Functional assessment should begin by establishing a reproducible molecular effect with the assays in Table 1, then selecting a model using the accession-specific residue mappings. These mappings nominate *flnba* L582P and Y1292C (A0AC58GU10), corresponding to human *FLNB* L582P and Y1294C, and *fndc1* F132S (A0AB40E856), corresponding to human *FNDC1* F108S, for consideration as knock-in alleles. The expressed transcript and residue identity should first be confirmed in the experimental strain. Because *flnba* and *flnbb* may have overlapping functions, paralogue expression should be assessed when interpreting a single-locus phenotype. Redundancy and compensation were not tested here. Any subsequent combined perturbation would require matched controls to distinguish the candidate allele’s effect from reduced total filamin dosage. In that model, shear-wave velocity would provide a mechanical readout, absolute thickness and thickness heterogeneity would describe tissue organization, and longitudinal coronal and sagittal angles would track morphology. Measurements should remain linked to identified animals and genotype controls. Simple baselines would determine whether a structural descriptor adds predictive information. Human *FNDC1* Y1880 corresponds to phenylalanine in the retrieved fish sequences, favouring a human-cell or humanized-sequence context for Y1880C. In the untreated 10-day source groups, the pooled velocity standard deviation was 0.566 m s^−1^ and the observed *sspo*–WT difference was 1.363 m s^−1^. Using the smaller hypothetical shifts of 0.25 and 0.50 m s^−1^ gives approximately 82 and 22 independent animals per group, respectively, under the stated planning assumptions. These scenarios do not predict a missense allele’s effect. Allele-specific pilot variance, developmental age, clutch and tank structure, and attrition would need to inform the final design.

### Limitations and Validation Priorities

The stability screen remains sensitive to method, threshold and WT-reference choice. The joint lists showed no excess overlap at 0.5, 1.0 or 1.5 kcal mol^−1^; combining the two stability thresholds therefore demonstrated no additional selection signal conditional on their marginal counts. DynaMut2 was applied only to the shortlist. Full-set AlphaMissense annotation showed exploratory enrichment, but neither this concordance nor the stability intersection validates clinical or functional relevance. Folding measurements and gene-specific assays are needed to validate molecular effects [15,19]. The screen does not establish allele-specific AIS risk or a three-method ranking of all 56 eligible substitutions.

The shortlisted human alleles were not tested in the fish mechanical datasets, leaving their direct effects on spinal tissue mechanics unvalidated. Refitting the source measurements is not independent replication, and the CSF-associated fish models sample only some scoliosis mechanisms.

The post-onset measurements cannot determine whether mechanics preceded curvature or changed in response to it. Notochord vacuoles contribute to vertebral compression responses during growth [41]; developmental factors could therefore influence both tissue mechanics and shape. The internal regression improves on the training-mean baseline but retains a 24.6° RMSE. With only 20 animals, performance estimates can be unstable [70]. Prospective assessment requires earlier mechanics, later plane-specific angles, baseline curvature and developmental covariates. Individual geometry would also need to be measured in those animals before testing mediation.

## 5. Conclusions

The stability analyses nominate four substitutions with unequal computational support. Three retain the exploratory classification across WT-reference definitions, while *FNDC1* Y1880C remains reference-sensitive and directionally discordant in DynaMut2. Only Y1294C and F108S exceeded the original threshold in the targeted third-method check.

Geometry transfer was assessed in two treated-group means after calibration on two untreated means and did not improve on the constant untreated-WT baseline. The velocity–angle analysis concerns 20 mutant fish measured after curve onset and retains a leave-one-animal-out error of 24.6°. Its source-defined outcome mixes the most severe sagittal or coronal angle. These observations do not establish prospective prediction, clinical utility or direction of causation. The molecular shortlist and readout-specific controls provide a basis for staged functional tests in candidate-specific models.

## Figures and Tables

**Table 1:** Four nominated substitutions, computational support and suggested functional tests. Scores T/E/P denote ThermoMPNN, EvoEF with globally repaired wild type (WT), and EvoEF with paired locally optimized WT, respectively, in kcal mol^−1^ (positive means predicted destabilization). D gives the targeted DynaMut2 estimate after sign reversal to positive destabilization. AM denotes AlphaMissense, a pathogenicity score rather than a stability energy. Combined Annotation Dependent Depletion (CADD) denotes GRCh38 v1.7 Phred-scaled scores; phyloP denotes the hg38 100-vertebrate nucleotide score. Frequencies are Genome Aggregation Database (gnomAD) r4 exome allele frequencies (AF); pLDDT denotes the predicted Local Distance Difference Test confidence score; VUS denotes a variant of uncertain significance, with one submitter for each returned ClinVar record. An unreturned record is not a measured zero or a benign classification. Among these four candidates, only Y1294C and F108S exceed 1 in all three methods; this does not establish experimental validity.

Candidate	Scores T/E/P; D	AM/CADD/ phyloP	gnomAD AF	ClinVar	Coverage	Suggested First Test
*FLNB* L582P	1.84/1.74/1.78; D: 0.219	0.9445 27.5/7.939	Not found	No match returned	pLDDT 84.4; no assembly	Domain stability, abundance and actin/partner binding
*FLNB* Y1294C	1.82/1.81/1.38; D: 1.557	0.7916 26.2/4.539	$3.42\;\times \;10^{-6}$	VUS	pLDDT 86.8; no assembly	Domain stability, abundance and gene-specific interactions
*FNDC1* F108S	1.49/1.76/1.56; D: 2.090	0.9715 27.5/3.407	$8.94\;\times \;10^{-6}$	VUS	pLDDT 89.0; no assembly	Domain stability, localization and mineralization assays
*FNDC1* Y1880C	1.39/2.76/0.78; D: −0.301	0.9441 25.4/6.473	$2.10\;\times \;10^{-4}$	VUS	pLDDT 90.7; no assembly	Lower priority: Test reference- and method-sensitive stability in human cells

## Data Availability

Supplementary Data S1 supplies all 121 variant records, 102 scored substitutions, eligibility/exclusion decisions, candidate annotations and sensitivity results. Supplementary Data S2 and S3 supply the 35-protein panel and 70-entry assembly inventory; Supplementary Data S4 supplies extracted animal observations and statistical results. Original measurements and structures retain their source attribution [46,49,59]. The complete code and data are publicly available at https://doi.org/10.5281/zenodo.23906016. No new human or animal experiments were performed.

## Supplementary Materials

The following supporting information can be downloaded at: https://www.mdpi.com/article/10.3390/biology15212048/s1. Supplementary Notes S1–S6 cover assembly sensitivity and coverage; units and cross-scale correspondence; stability-reference sensitivity; variant provenance, selection and stability sensitivity; baselines, sampling uncertainty and regression diagnostics; and protein-panel selection and assembly inventory. Figures S1–S3 show experimental structural contacts, regression-model diagnostics, and structural localization of candidates with discordant stability predictions. Tables S1–S3 provide variant records and disposition, the 35-protein panel and archived entry selection, and the 70-entry assembly-1 inventory. Supplementary Data S1–S4 provide variant records and sensitivity analyses, the protein panel, the assembly inventory, and extracted animal observations and statistical results. The cited sources include [12,46,49,68].

## Abbreviations

The following abbreviations are used in this manuscript:

AF	Allele frequency
AIS	Adolescent idiopathic scoliosis
AM	AlphaMissense score
API	Application programming interface
CADD	Combined Annotation Dependent Depletion
CSF	Cerebrospinal fluid
ECM	Extracellular matrix
gnomAD	Genome Aggregation Database
HC3	Jackknife heteroscedasticity-consistent covariance estimator
LTBP1	Latent transforming growth factor-β binding protein 1
NACET	N-acetyl-L-cysteine ethyl ester
NMR	Nuclear magnetic resonance
PDB	Protein Data Bank
PDZ	PSD-95/Dlg/ZO-1
pLDDT	Predicted Local Distance Difference Test
RCSB	Research Collaboratory for Structural Bioinformatics
RMSE	Root-mean-square error
SHA-256	256-bit Secure Hash Algorithm
SIFTS	Structure Integration with Function, Taxonomy and Sequences
TAZ	Transcriptional coactivator with PDZ-binding motif
TGF-β	Transforming growth factor-β
UCSC	University of California, Santa Cruz
VUS	Variant of uncertain significance
WT	Wild type
YAP	Yes-associated protein
