# Supplementary material for: Candidate Variant Prioritization and Tissue-Mechanics Endpoints for Functional Validation in Scoliosis: A Computational Reanalysis
Source: Biology (Basel). 2026 Nov 9;15(21):2048. doi: 10.3390/biology15212048 (PMC13761410; doi:10.3390/biology15212048)
Supplement: Supplementary file 1 [file biology-15-02048-s001.zip › supplementary_information.pdf]

Supplementary Information

Supplementary Note S1: assembly sensitivity and coverage

The assembly contact network in Figure S1 assesses structural coverage. The survey processed 70 selected assembly-1 coordinate files without recorded failures. Fifty-three entries generated contact-residue-qualified chain-pair records. The primary area threshold retained 162 chain pairs; restricting both partner labels to the panel and requiring a single UniProt accession per chain yielded 54 chain pairs and 14 unordered protein pairs, six heterotypic. Twelve panel-labelled chain pairs from four type-I/type-IX collagen chimeric constructs were excluded because whole-chain buried area cannot be assigned specifically to type-I collagen. Their contact records are retained in the compact package; its exported construct-exclusion table identifies the excluded pairs. These counts do not represent independent biological replicates.

Threshold tabulations below were restricted to the initially selected, panel-mapped chain pairs. The initial requirement of at least three contacting residues on each partner within 5 Å was not recomputed for each distance. The displayed distance filter required at least three atom-pair contacts at the indicated cutoff. A 50 $Å^2$ area cutoff therefore did not add interfaces excluded by the initial 100 $Å^2$ primary screen. These checks describe dependence within the retained set rather than an unbiased rescreening of all possible pairs.

Area cutoff	Heterotypic pairs at contact cutoff			Chain pairs	Protein pairs
($Å^2$)	4 Å	5 Å	6 Å	(5 Å)	(5 Å)
50	6	6	6	54	14
100	6	6	6	54	14
200	6	6	6	54	14
500	5	5	5	47	11

Increasing sphere sampling from 240 to 960 points per atom changed buried areas by at most 0.681% for ten representative chain pairs from 7NWL, 5CTD and 9R3S. All ten retained their primary-interface classification. The 5CTD pairs remain a numerical surface-sampling check, but their chimeric chains were excluded from the protein-specific network. This numerical check did not test biological relevance or structural completeness.

Only FLNB M1803L and R199Q among the source substitutions had exact reference-residue and position coverage in the selected assemblies. They were outside retained interfaces. Neither belongs to the four stability-prioritized substitutions, all of which lacked exact assembly coverage. The small covered set cannot establish an absence of interface effects. No interface result was assigned to a residue absent from experimental coordinates.

The heterotypic contacts connected collagen, fibronectin and integrin components within the selected structures. A short COL1A1 peptide bound to the FN1 type-I domain pair 8–9 in 3GXE had a mean one-sided buried area of 506 $Å^2$; this deposited complex is described in the primary study by Erat et al.[1]. FN1–ITGA5 and FN1–ITGB1 averaged 540 and 411 $Å^2$, respectively, across chain pairs represented in two assemblies each. ITGA5–ITGB1 averaged 1,575 $Å^2$ across chain pairs in five assemblies. The COL1A1–COL1A2 contact averaged 1,042 $Å^2$ across two chain pairs in the deposited peptide construct 8YV3. Its assembly contains one COL1A1-derived and two COL1A2-derived peptides, rather than the usual native type-I collagen stoichiometry. The separate contact between the LTBP1 TB3 domain and latent TGFB1 proprotein in 9R3S had an area of 1,022 $Å^2$. These descriptive areas quantify buried surfaces in deposited conformations; they are not binding energies or measurements of force transmission.

Supplementary Note S2: units and cross-scale correspondence

Human variant records, deposited assemblies and animal measurements originate from separate source studies. No shared identifier traces a prioritized human substitution into the zebrafish tissue measurements. Assembly contacts did not enter the stability-prioritization rule, tissue calibration or individual curvature regression. Network diagrams therefore indicate structural contacts rather than arrows in a causal pathway.

The 209 shear-wave observations belong to source comparisons that were analysed separately. They were not summed with the other animal records to infer a unique-animal total. The geometry analysis used 140

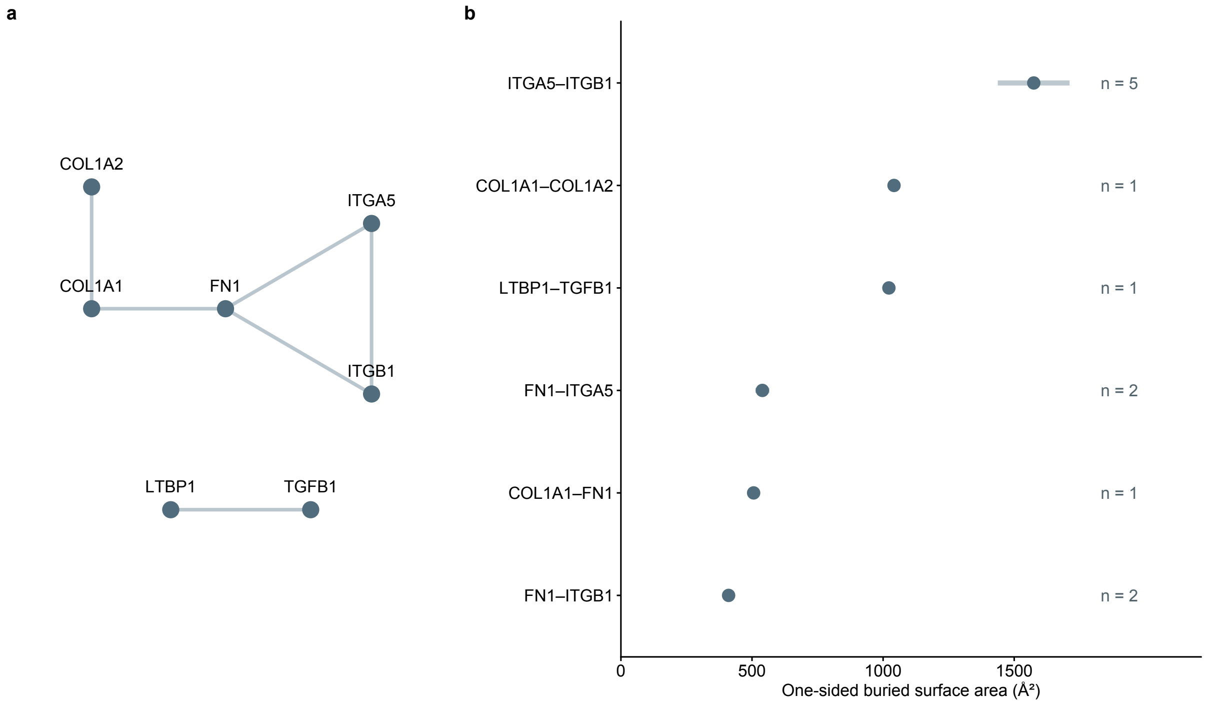

Figure S1. **Experimental structural contacts in the selected panel.** (**a**) Six heterotypic pairs; the undirected diagram is not a physical complex or causal pathway. (**b**) Mean one-sided buried areas, with observed minima/maxima rather than confidence intervals. Labels count distinct PDB entries; chain pairs are not independent biological replicates. Single-entry contacts derive from 3GXE (COL1A1 peptide–FN1 domains 8–9), 9R3S (LTBP1 TB3–latent TGFB1) and 8YV3 (collagen-derived peptides, with one COL1A1 and two COL1A2 chains). Multi-accession chimeric constructs were excluded from protein-specific areas. Areas describe deposited conformations, without measuring affinity or mechanical importance. None of the four shortlisted variants is covered.

segments with paired maximum and minimum thickness measurements. Published animal totals were 8, 9, 6 and 7 in untreated wild-type (WT), untreated mutant, treated WT and treated mutant groups, but segment-to-animal assignments were unavailable. Segment-weighted means were retained without pseudoreplicated animal-level intervals.

The redox analysis used 23 animals, with three regions of interest averaged within each animal. Group sizes were 3, 8, 3 and 9; the corresponding mechanical groups comprised 15 animals each in a separate cohort. The 20 velocity/Cobb-angle pairs supported leave-one-animal-out internal validation. They did not provide an external cohort, prospective measurements or individual geometry-to-curvature mediation.

Supplementary Note S3: stability-reference sensitivity

The primary EvoEF estimates subtract the globally repaired WT energy, following the published EvoEF workflow. The alternative subtracts the paired WT energy after local repacking of the mutation site and its neighbourhood. Both use the same mutant coordinates and positive destabilization convention.

Substitution	ThermoMPNN	EvoEF: repaired WT	EvoEF: paired WT
	Predicted $\Delta\Delta G$ (kcal mol^{-1})		
FLNB L582P	1.84	1.74	1.78
FLNB Y1294C	1.82	1.81	1.38
FNDC1 F108S	1.49	1.76	1.56
FNDC1 Y1880C	1.39	2.76	0.78

Three of the four main-reference candidates retain both-method predictions above 1 kcal mol^{-1} under the alternative reference. No additional candidate crosses both thresholds among the 56 primary-eligible variants. Y1880C is reference-sensitive and shows less consistent computational support than the other three. This comparison tests reference choice, not experimental stability or pathogenicity. Model-level inputs, all 102 scored variants and both reference definitions accompany Supplementary Data S1 and the reproducibility archive. The v2 code/data supplement additionally contains the recovered historical coordinates for all 56 primary-eligible substitutions (60 model–variant rows and 138 PDB files). The manifest links each scored substitution/conformer to its original WT, globally repaired WT, paired locally optimized WT and mutant coordinates, with SHA256 values and residue-identity checks. No historical structure was replaced by a newly generated surrogate. The complete offline replay and all-56 structure checks passed. The archive includes an MIT license for original project code and documentation and separate third-party notices, and is available as version v2 at `https://doi.org/10.5281/zenodo.23906016`.

Supplementary Note S4: Variant provenance, selection and stability sensitivity

Supplementary Data S1 provides all 121 unique gene–protein-substitution records, their original study and phenotype labels, the canonical mapping, and a record-specific analysis status. The 121 records comprise 3 COL11A1, 26 COL11A2, 9 FBN1, 22 FLNB and 61 FNDC1 substitutions. Study observations of the same gene and protein substitution were collapsed with all source labels retained; this is not 121 independent patients or cohorts. Of these, 19 were not scored: ten had ambiguous isoform mappings, one had a reference-residue mismatch, two were non-missense consequences, and six FBN1 substitutions lacked sequence-matched experimental coordinates. Of the 102 scored substitutions, 12 tentative isoform mappings and 34 AlphaFold sites with pLDDT below 70 were excluded from the primary comparison, leaving 56 (2 COL11A2, 2 FBN1, 17 FLNB and 35 FNDC1). The 46 excluded predictions remain available in the complete prediction table.

Cutoff	ThermoMPNN	EvoEF	Observed	Expected	$P(K \geq k)$
0.5	27	21	12	10.125	0.224
1.0	16	14	4	4.000	0.624
1.5	8	8	2	1.143	0.320

Cutoffs are in kcal mol^{-1} and use strict > inequalities in both methods. Under an independent-label null conditional on the marginal numbers above cutoff, $K \sim$ Hypergeometric$(56, n_T, n_E)$ and $\mathbb{E}[K] = n_T n_E/56$. At the primary cutoff, 16 ThermoMPNN and 14 EvoEF predictions exceed 1, giving four expected overlaps and four observed overlaps ($P(K \geq 4) = 0.624$). A within-gene random-label null gives an expected overlap of 4.191. Thus the screen does not demonstrate excess concordance above chance. These calculations describe score-list overlap and neither estimate a biological false-discovery rate nor validate the shortlisted substitutions. At 0.5 the candidate list expands to 12; at 1.5 it contracts to FLNB L582P and Y1294C. The complete lists and both EvoEF reference definitions are supplied in Supplementary Data S1.

The 1 kcal/mol threshold is an exploratory prioritization convention, not a clinical decision boundary. Its biological interpretation is limited by tool disagreement, score calibration and omitted molecular context. No threshold was selected by optimizing a downstream animal phenotype. The four main-reference candidates remain hypotheses requiring direct measurements; FNDC1 Y1880C is lower priority because its paired-reference EvoEF estimate is only 0.78 kcal/mol.

Candidate annotations were retrieved on 28 October 2026 using exact transcript/protein consequences and genome-build-specific alleles. ClinVar VCV000346339.5 (FLNB Y1294C), VCV003851043.1 (FNDC1 F108S) and VCV002457140.3 (FNDC1 Y1880C) each reported uncertain significance with criteria from one submitter. The first concerned FLNB-related spectrum disorders; the FNDC1 records did not specify a condition. No exact FLNB L582P record was retrieved by the gene/protein/HGVS searches. None of these outcomes establishes an AIS-specific clinical classification. Queries included both one-letter and three-letter amino-acid notation.

The matching GRCh38 alleles were FLNB L582P 3-58105214-T-C, FLNB Y1294C 3-58124488-A-G, FNDC1 F108S 6-159200014-T-C and FNDC1 Y1880C 6-159271396-A-G. The L582P source allele (GRCh37 3-58090941-T-C) was mapped independently by Ensembl assembly conversion and current RefSeq HGVS annotation; both gave the same GRCh38 allele and Leu582Pro consequence. In the gnomAD r4 dataset, exome allele counts/allele numbers were respectively not returned, 5/1461884, 13/1453724 and 307/1460294. Their available exome allele frequencies were 3.4202×10^{-6}, 8.9426×10^{-6} and 2.1023×10^{-4}. L582P returned "Variant not found", which is not a measured zero frequency. Genome cohort counts and allele numbers are retained separately in Supplementary Data S1 and are not added to exome counts. The original FLNB study's historical database-absence code is not substituted for a current frequency.

For FNDC1 Y1880C, the European ancestry of the source cohort makes the non-Finnish European frequency particularly relevant. The gnomAD r4 ancestry-specific exome counts below were retrieved on 3 November 2026. AC/AN denotes alternate allele count/number of called alleles; a returned AC of zero is distinguished from an unavailable cohort. Genome counts are archived separately, without summing the two cohorts.

Genetic ancestry group	AC	AN	Exome AF
African/African American	0	33,446	0
Admixed American	4	44,590	8.9706×10^{-5}
Ashkenazi Jewish	1	26,082	3.8341×10^{-5}
East Asian	0	39,654	0
European (Finnish)	32	53,330	6.0004×10^{-4}
European (non-Finnish)	253	1,111,348	2.2765×10^{-4}
Middle Eastern	0	5,768	0
South Asian	0	85,752	0
Remaining	17	60,324	2.8181×10^{-4}

The overall exome count was 307/1,460,294, including one non-Finnish European homozygote. These observations do not support interpreting Y1880C as a highly penetrant severe-AIS allele. They do not estimate penetrance or exclude a lower-penetrance contribution, which would require allele-specific, ancestry-matched association and functional evidence.

Archived AlphaMissense scores for L582P, Y1294C, F108S and Y1880C were 0.9445, 0.7916, 0.9715 and 0.9441, respectively. These sequence-level pathogenicity scores complement the stability screen but do not constitute a third thermodynamic calculation or clinical validation. The revision completed AlphaMissense annotations for all 56 eligible substitutions, including FBN1 N2178K (0.5569) and N280T (0.1697). Nineteen exceeded the released likely-pathogenic cutoff of 0.564: two of 17 FLNB and 17 of 35 FNDC1 substitutions, with none among the two FBN1 and two COL11A2 substitutions. All four shortlisted substitutions exceeded the cutoff. The unstratified expected overlap was $4 \times 19/56 = 1.357$, with an exact upper-tail hypergeometric $P = 0.010553$. Conditioning separately on two shortlisted FLNB and two shortlisted FNDC1 substitutions gave expected overlap $2 \times 2/17 + 2 \times 17/35 = 1.206723$ and exact upper-tail

$P = 0.001681$. The ThermoMPNN and EvoEF marginal lists contained 11/16 and 7/14 AlphaMissense-positive substitutions, respectively. These retrospective, unadjusted annotation comparisons do not establish superiority of the intersection, a thermodynamic consensus or a clinical benchmark. The complete 56-row scores and reproducible calculations are provided in the code/data supplement. The exact data directory is `review_revision/variant_data/data/`, containing `alphamissense_all56.csv`, `candidate_annotations_review.csv` and `FNDC1_Y1880C_gnomAD_ancestry.csv`; raw retrievals and the verification script accompany them. The pre-existing Supplementary Data S1–S4 workbook retains its historical source values unchanged. The original FNDC1 Supplementary Table S7 reported CADD PHRED scores of 29.3 for F108S and 26.2 for Y1880C; the latter score was verified against the source workbook. These historical source scores are distinct from uniform GRCh38 CADD v1.7 queries performed on 3 November 2026. Current CADD v1.7 PHRED scores for FLNB L582P, FLNB Y1294C, FNDC1 F108S and FNDC1 Y1880C were 27.5, 26.2, 27.5 and 25.4; the corresponding UCSC hg38 100-vertebrate phyloP nucleotide scores were 7.93862, 4.53898, 3.40749 and 6.47250. Positive phyloP scores indicate evidence of nucleotide conservation, without establishing protein function or pathogenicity. Raw queries, exact alleles, versions and retrieval records are supplied in the code/data supplement. Missing score fields are labelled explicitly rather than imputed.

For experimental translation, human UniProt O75369 and Q4ZHG4 sequences were aligned to all retrieved Danio rerio flnba, flnbb and fndc1 entries using global BLOSUM62 alignments with gap-open/extension scores of $-10/-0.5$ and $-12/-1$. For flnba A0AC58GU10 (and its second retrieved isoform), human FLNB L582 and Y1294 aligned to L582 and Y1292 under both settings; local 31-residue identities were 77.4% and 67.7%. FNDC1 F108 aligned to F132 in fndc1 A0AB40E856 (54.8% local identity). Human FNDC1 Y1880 aligned instead to F1667 (and F1638 in the other retrieved fndc1 isoform), so the tyrosine identity was not conserved. These are sequence correspondences in specified database entries. Confirmed molecular effects, isoform expression and paralog compensation must be assessed before knock-in experiments. The non-conserved Y1880 site calls for a human-cell or humanized-sequence system rather than assuming an equivalent fish tyrosine substitution. No knock-in fish or new animal measurements were generated.

Targeted DynaMut2 assessment of the original four candidates

DynaMut2 predictions were obtained for all four candidates. The input was the publicly available full-length WT AlphaFold coordinate model, chain A; returned chain and substitution identities and finite values were verified against the submitted sequence. Raw DynaMut2 negative values denote destabilization. The second score column reverses this sign to use the positive-destabilization convention.

Candidate	Raw DynaMut2	Positive destabilization	Job ID
FLNB L582P	−0.219	+0.219	179314499131
FLNB Y1294C	−1.557	+1.557	179314498378
FNDC1 F108S	−2.090	+2.090	179314498061
FNDC1 Y1880C	+0.301	−0.301	179314498804

Scores are in $\mathrm{kcal\,mol^{-1}}$. Only Y1294C and F108S exceed 1 on the positive-destabilization convention. L582P has a smaller predicted effect; Y1880C has the opposite sign and remains lower priority. This is a targeted sensitivity assessment rather than a third-method screen of all 56 variants. The original two-method candidate definition was retained, and method agreement does not constitute experimental validation. Supplementary Data S1 retains raw and sign-harmonized values; input hashes, timestamps and unmodified responses are included in the reproducibility archive.

Complete record-level inventory

Supplementary Data S1 retains every original study, phenotype and annotation field. The compact inventory below provides all 121 records, including excluded entries. Status codes are P, primary eligible; L, low pLDDT; T, tentative mapping; A, ambiguous isoform; C, no sequence-matched experimental coverage; N, non-missense; and R, reference mismatch. A dash means no accepted canonical mapping. Source keys are defined immediately below; repeated study observations are retained in the machine-readable source fields.

S1: Haller2016 Figure3A DOI:10.1093/hmg/ddv463

S2: Haller2016 Figure3A DOI:10.1093/hmg/ddv463;Haller2016 Figure3A DOI:10.1093/hmg/ddv463

S3: PMC10945706 Supplement2; Yu et al., eLife (2024), doi:10.7554/eLife.89762

S4: PMC12187536 Supplement Table S6

S5: PMC12187536 Supplement Table S6;PMC12187536 Supplement Table S7

S6: PMC12187536 Supplement Table S7

S7: PMC7279190 Table3

S8: PMC7279190 Table3;PMC7279190 Table3

S9: PMC7279190 Table3;PMC7279190 Table3;PMC7279190 Table3

Table S1. All variant records and their analytical disposition. Original protein numbering is preserved alongside canonical numbering.

ID	Gene	Source variant	Canonical	Source	Status
V001	COL11A1	A1365T	A1353T	S3	L
V002	COL11A1	G1132S	G1120S	S3	L
V003	COL11A1	V1283L	V1271L	S3	L
V004	COL11A2	G286S	–	S1	A
V005	COL11A2	S288P	–	S1	R
V006	COL11A2	A361V	–	S1	A
V007	COL11A2	L364I	–	S1	A
V008	COL11A2	A374T	–	S1	A
V009	COL11A2	G560C	–	S1	A
V010	COL11A2	G620R	–	S1	A
V011	COL11A2	P702L	–	S1	A
V012	COL11A2	P865H	–	S1	A
V013	COL11A2	P1005H	–	S1	A
V014	COL11A2	P1056L	–	S1	A
V015	COL11A2	A145G	A145G	S1	P
V016	COL11A2	A867V	A974V	S1	T
V017	COL11A2	E314*	E314*	S1	N
V018	COL11A2	N1042S	N1149S	S1	T
V019	COL11A2	P1427S	P1534S	S1	T
V020	COL11A2	P324S	P431S	S1	T
V021	COL11A2	P709H	P816H	S1	T
V022	COL11A2	R913Q	R1020Q	S1	T
V023	COL11A2	R1452Q	R1559Q	S1	T
V024	COL11A2	R1489Q	R1596Q	S1	T
V025	COL11A2	R1617W	R1724W	S1	T
V026	COL11A2	R180H	R180H	S2	P
V027	COL11A2	R390C	R497C	S2	T
V028	COL11A2	S305L	S305L	S1	T
V029	COL11A2	S757Y	S864Y	S1	T
V030	FBN1	M1690Kfs*12	–	S4	N
V031	FBN1	D1155N	D1155N	S4	C
V032	FBN1	L1405R	L1405R	S4	C
V033	FBN1	N2178K	N2178K	S4	P
V034	FBN1	N280T	N280T	S4	P
V035	FBN1	N703H	N703H	S4	C
V036	FBN1	Q2467R	Q2467R	S4	C
V037	FBN1	R976H	R976H	S4	C
V038	FBN1	V2868I	V2868I	S4	C
V039	FLNB	A1059T	A1059T	S7	P
V040	FLNB	A2282T	A2282T	S7	L
V041	FLNB	A2315T	A2315T	S7	L
V042	FLNB	E1144Q	E1144Q	S7	P
V043	FLNB	L582P	L582P	S7	P
V044	FLNB	M1803L	M1803L	S9	P

ID	Gene	Source variant	Canonical	Source	Status
V045	FLNB	P1958S	P1958S	S7	L
V046	FLNB	R1605H	R1605H	S7	P
V047	FLNB	R1914W	R1914W	S8	L
V048	FLNB	R199Q	R199Q	S7	P
V049	FLNB	R2003H	R2003H	S7	P
V050	FLNB	R2374H	R2374H	S7	P
V051	FLNB	R470Q	R470Q	S7	P
V052	FLNB	R566L	R566L	S7	P
V053	FLNB	R566W	R566W	S7	P
V054	FLNB	S1371L	S1371L	S7	P
V055	FLNB	S2503G	S2503G	S7	L
V056	FLNB	T1575M	T1575M	S7	P
V057	FLNB	T2166M	T2166M	S7	P
V058	FLNB	T770I	T770I	S7	P
V059	FLNB	V1195M	V1195M	S7	P
V060	FLNB	Y1294C	Y1294C	S7	P
V061	FNDC1	A1220T	A1220T	S6	L
V062	FNDC1	A612T	A612T	S6	L
V063	FNDC1	A637S	A637S	S6	L
V064	FNDC1	A76T	A76T	S6	P
V065	FNDC1	D1359N	D1359N	S6	P
V066	FNDC1	D1523G	D1523G	S6	L
V067	FNDC1	D261G	D261G	S6	P
V068	FNDC1	D561V	D561V	S6	L
V069	FNDC1	D60N	D60N	S6	P
V070	FNDC1	D793N	D793N	S6	L
V071	FNDC1	E1599G	E1599G	S6	P
V072	FNDC1	E649G	E649G	S6	L
V073	FNDC1	F108S	F108S	S6	P
V074	FNDC1	G442S	G442S	S6	P
V075	FNDC1	G628V	G628V	S6	L
V076	FNDC1	G744E	G744E	S6	L
V077	FNDC1	H1671L	H1671L	S6	P
V078	FNDC1	H631Y	H631Y	S6	L
V079	FNDC1	I75V	I75V	S6	P
V080	FNDC1	K374E	K374E	S6	P
V081	FNDC1	K503E	K503E	S5	L
V082	FNDC1	M1528K	M1528K	S6	P
V083	FNDC1	M271V	M271V	S6	P
V084	FNDC1	N1531S	N1531S	S6	L
V085	FNDC1	P1006H	P1006H	S6	L
V086	FNDC1	P1683T	P1683T	S6	L
V087	FNDC1	P401L	P401L	S6	P
V088	FNDC1	P569Q	P569Q	S6	L
V089	FNDC1	Q800K	Q800K	S6	L
V090	FNDC1	R121C	R121C	S4	P
V091	FNDC1	R1233L	R1233L	S6	L
V092	FNDC1	R1308C	R1308C	S6	L
V093	FNDC1	R1496W	R1496W	S6	L
V094	FNDC1	R1586S	R1586S	S6	L
V095	FNDC1	R163C	R163C	S6	P
V096	FNDC1	R1660C	R1660C	S6	P
V097	FNDC1	R1789C	R1789C	S4	P
V098	FNDC1	R1793Q	R1793Q	S6	P
V099	FNDC1	R215W	R215W	S6	P
V100	FNDC1	R318W	R318W	S6	P
V101	FNDC1	R319C	R319C	S6	P
V102	FNDC1	R44S	R44S	S6	P

ID	Gene	Source variant	Canonical	Source	Status
V103	FNDC1	R674C	R674C	S6	L
V104	FNDC1	R811W	R811W	S6	L
V105	FNDC1	S1570T	S1570T	S6	L
V106	FNDC1	S1639T	S1639T	S6	L
V107	FNDC1	S695L	S695L	S6	L
V108	FNDC1	S955N	S955N	S6	L
V109	FNDC1	T1779M	T1779M	S6	P
V110	FNDC1	T1817K	T1817K	S6	P
V111	FNDC1	T348M	T348M	S6	P
V112	FNDC1	T361I	T361I	S6	P
V113	FNDC1	T412N	T412N	S6	P
V114	FNDC1	V123A	V123A	S6	P
V115	FNDC1	V1664M	V1664M	S6	P
V116	FNDC1	V1730L	V1730L	S6	P
V117	FNDC1	V175I	V175I	S6	P
V118	FNDC1	V1796M	V1796M	S6	P
V119	FNDC1	V1798L	V1798L	S6	P
V120	FNDC1	V299M	V299M	S6	P
V121	FNDC1	Y1880C	Y1880C	S5	P

Supplementary Note S5: Baselines, sampling uncertainty and regression diagnostics

A constant wild-type baseline for the geometry calibration

The geometry power law has two fitted parameters (scale and exponent) determined by exactly two untreated group means. Its agreement with those training means is therefore algebraic, with no residual degrees of freedom for estimating fit uncertainty. The two treated groups were retained as withheld groups. We compared the power law with a retrospectively specified constant prediction equal to the untreated wild-type mean squared velocity, $4.56368\,\mathrm{m^2\,s^{-2}}$. No treated-group value entered this baseline.

Withheld group	Observed G	Geometry $\widehat{G}$	WT baseline	Geometry error	WT error
WT + NACET	4.90434	4.51562	4.56368	−7.93%	−6.95%
Mutant + NACET	4.35185	4.74349	4.56368	+9.00%	+4.87%

Observed and predicted G values are in $\mathrm{m^2\,s^{-2}}$. Relative error is $100(\widehat{G}/G-1)$. Across the two withheld means, root-mean-square errors were 0.39018 for geometry and $0.28366\,\mathrm{m^2\,s^{-2}}$ for the WT baseline; mean absolute relative errors were 8.46% and 5.91%, respectively. The constant baseline had the smaller error in each group. The geometry result is compatible with a return towards the control state, without demonstrating predictive value beyond that return. Two withheld means do not support a reliable model-ranking test or a claim of general transferability.

The 140 geometry observations are segments from published totals of 8, 9, 6 and 7 animals; source records do not map individual segments to those animals. Segment-weighted means can be influenced by unequal numbers of segments per animal and within-animal correlation. Neither animal-weighted means nor cluster-resampled geometry intervals can be recovered. Segment-level resampling would understate uncertainty and was not performed. Likewise, the redox calibration is ecological: the redox groups contain 3, 8, 3 and 9 animals and the mechanical groups 15 each, from separate measurements without paired individual correspondence. Its apparent transfer differences are exploratory comparisons of two means.

Uncertainty in internal Cobb-angle prediction

The source data provide 20 paired observations from 21-day-post-fertilization *sspo* mutants after curve onset. The source endpoint is the most severe Cobb angle in either the sagittal or coronal plane, rather than a uniformly coronal human clinical Cobb endpoint. The fitted ordinary-least-squares slope was 26.0926° per

$\mathrm{m\,s^{-1}}$, with fitted $R^2 = 0.697695$. The leave-one-animal-out RMSE was 24.6020°, and the training-mean baseline RMSE was 42.3290°. The unconstrained linear prediction was retained, including any negative values.

To assess finite-sample instability, 20,000 nonparametric case-bootstrap samples of 20 animals were drawn with replacement (seed 261028). Each original source row is an animal identity within this paired dataset. For each bootstrap sample, prediction for a held-out identity excluded every copy of that identity from the training set; the regression was refitted using the remaining animals with their bootstrap multiplicities as weights. The baseline used the mean of the same training observations. Squared errors were then averaged using held-out multiplicities before taking the square root. This procedure resamples animals and refits the prediction algorithm; it does not treat the 20 overlapping folds as independent replicates or allow copies of the same animal into both training and test data.

The 2.5th–97.5th percentiles were 17.48°–34.97° for regression RMSE and 30.73°–53.89° for baseline RMSE. For the paired difference (baseline minus regression RMSE), the point estimate was 17.73° and the percentile interval 2.54°–31.60°. The regression mean absolute error was 20.44°, with percentile interval 13.92°–30.20°. All 20,000 resamples yielded finite estimates. These are internal resampling intervals, not individual prediction intervals, external-validation results or guaranteed nominal-confidence bounds. Resampling with replacement reduces the number of distinct animals (median 13; central 95% range 10–16), so the intervals also reflect instability of smaller distinct training samples. They must not be interpreted as precise population-level accuracy estimates.

Residual, distributional and influence checks

We examined residual-versus-fitted, normal quantile–quantile and Cook's-distance plots for the Cobb regression and the genotype-by-NACET velocity model (Figure S2). The latter uses the 60 animals shown in Figure 5f of the source study (15 per group), with four fitted coefficients. Shapiro–Wilk tests were applied to residuals as descriptive distributional checks; studentized Breusch–Pagan tests assessed variance patterns. These tests have limited power in small samples, and non-rejection does not validate a model.

Model	n	Residual df	Shapiro W	Shapiro P	Breusch–Pagan P
Cobb angle on velocity	20	18	0.97989	0.93267	0.70754
Velocity on genotype × NACET	60	56	0.96064	0.05056	0.22572

The velocity-model quantile plot shows tail deviations, with a Shapiro–Wilk result close to 0.05. HC3 intervals assess sensitivity to unequal error variances; the diagnostic tests do not establish normally distributed errors. Using residual-degrees-of-freedom t critical values, the Cobb slope HC3 interval is 16.83°–35.36° per $\mathrm{m\,s^{-1}}$ ($P = 1.34 \times 10^{-5}$). The velocity genotype-by-NACET interaction remains $-1.50756\,\mathrm{m\,s^{-1}}$, with HC3 interval -2.01462 to $-1.00050\,\mathrm{m\,s^{-1}}$ ($P = 1.79 \times 10^{-7}$). These calculations test sensitivity of coefficient inference; they do not resolve selection, causality or external validity.

The largest Cook's distance is 0.38873 for Cobb angle and 0.21973 for velocity; one of 20 and three of 60 observations exceed the descriptive $4/n$ reference, respectively. This reference flags observations for inspection and is not an exclusion rule. No animal was discarded. Deleting each Cobb animal in turn gives slopes of 24.57°–29.21° per $\mathrm{m\,s^{-1}}$ and fitted R^2 values of 0.623–0.763. These sensitivities support an association within the source dataset while underscoring its small-sample nature. The post-onset, mixed-plane endpoint, remaining prediction error and possible reverse direction between existing curvature and measured mechanics preclude a claim of clinical prognosis or causal mediation. For future allele-specific studies, the untreated 10-day source groups (15 WT and 15 *sspo* animals) gave means of 2.08528 and $3.44811\,\mathrm{m\,s^{-1}}$, standard deviations of 0.48028 and $0.63989\,\mathrm{m\,s^{-1}}$, and pooled variance $0.320068955\,\mathrm{m^2\,s^{-2}}$. Base-R two-sided two-sample t-test planning at $\alpha = 0.05$, power 0.80 and equal group sizes gave 81.3614 and 21.1023 animals per group for assumed differences of 0.25 and $0.50\,\mathrm{m\,s^{-1}}$, rounded upward to 82 and 22. These smaller shifts are illustrative scenarios, not measured missense effects. Attrition, clutch/tank clustering and a different developmental stage may increase the required sample; an allele-specific pilot should inform the final design. The calculation script and source-row checks are supplied in the code/data supplement.

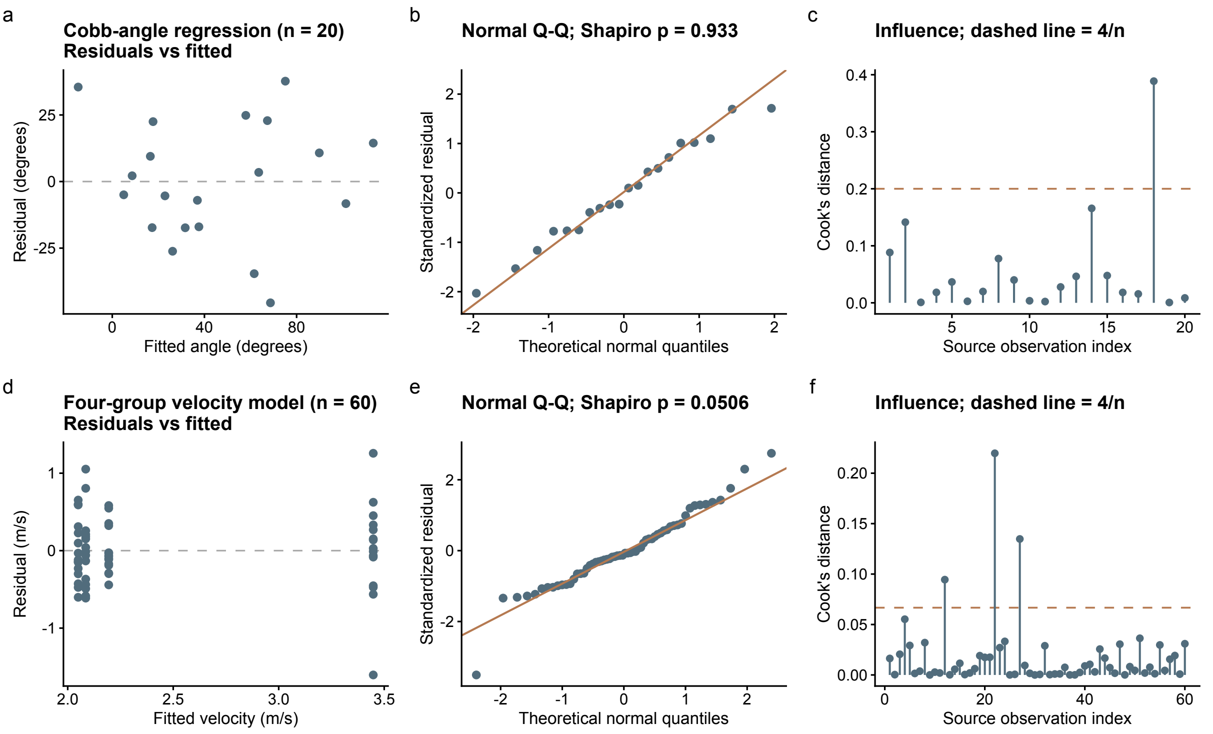

Figure S2. **Regression model diagnostics.** (**a**–**c**) Cobb angle on velocity in 20 *sspo* mutants after curve onset. (**d**–**f**) Velocity on genotype, NACET and their interaction in 60 animals (15 per group). Panels show residuals versus fitted values, normal quantile–quantile plots of standardized residuals, and Cook's distances. The dashed reference in influence panels is $4/n$ and does not define an exclusion criterion. Each point is one source-reported animal within its analysis; no cross-sheet animal correspondence is assumed. No observations were removed.

Supplementary Note S6: protein-panel selection and assembly inventory

The 35-protein panel was selected for structural context, rather than inferred from a statistically enriched gene set. It contains 12 initial genetics-motivated panel genes and 23 deliberately added matrix components, matrix receptors, remodelling enzymes, adhesion proteins or mechanosensing/signalling proteins. This broader context panel differs from the five genes represented in the 121-record variant atlas. Panel membership therefore does not establish disease association. Supplementary Data S2 supplies every gene, human UniProt accession, inclusion category and selected-entry list. RCSB was queried by exact UniProt accession. Entries required at least two protein entities or at least two polymer instances, and those with 180,000 or more deposited atoms were excluded. Eligible entries were ordered by the tuple (at least three protein entities, electron-microscopy method, better resolution, entry identifier), in descending order except for resolution through its negation; at most four entries were retained per gene. The union was 70 entries. This purposeful, capped survey is not an exhaustive or unbiased structural census. The archived per-gene rank and union are retained rather than regenerated against a changed database. Supplementary Data S3 supplies one row for each selected entry, including assembly 1, selecting gene(s) and accession(s), archived within-gene rank, accessions and panel genes actually mapped in processed coordinates, processed-residue counts, retained and excluded chain-pair counts, and separate coordinate, entry-metadata, assembly-metadata and SIFTS URLs. The frozen residue and interface tables provide coordinate-derived provenance for all 70 entries. Historical coordinate revision identifiers and acquisition timestamps are unavailable in the lightweight archive; the URLs identify repository records and must not be interpreted as immutable version identifiers. A fresh local revision snapshot was acquired for all 70 assembly-1 mmCIF files and, for each entry, entry metadata, assembly metadata, SIFTS accession mappings and residue XML (350 successful retrievals). The compact package supplies the official URLs, UTC retrieval records and SHA256 values with optional checked downloads, rather than duplicating these public objects. Supplementary Data S3 records available current revision information separately from the frozen historical analysis tables. Identity of atom coordinates to the historical scoring inputs has not been authenticated; these downloads do not retrospectively recover that snapshot.

Table S2. Complete 35-protein panel and archived entry selection. Genes without selected entries remain in the panel denominator.

Gene	UniProt	Inclusion basis	Selected entries
COL11A2	P13942	initial genetics-motivated panel	None
FBN1	P35555	initial genetics-motivated panel	1UZJ
FBN2	P35556	initial genetics-motivated panel	None
COL11A1	P12107	initial genetics-motivated panel	None
COL2A1	P02458	initial genetics-motivated panel	5OCX, 7NZE, 6NIX, 2SEB
COL3A1	P02461	initial genetics-motivated panel	7WWS, 7WWR, 4GYX, 7XAN
COL5A2	P05997	initial genetics-motivated panel	None
COL6A3	P12111	initial genetics-motivated panel	13BF, 9GTU, 6SNK
HSPG2	P98160	initial genetics-motivated panel	None
FNDC1	Q4ZHG4	initial genetics-motivated panel	None
FLNB	O75369	initial genetics-motivated panel	4B7L, 3FER, 5DCP
ADGRG6	Q86SQ4	initial genetics-motivated panel	21DU
FN1	P02751	matrix assembly	9P6S, 7NWL, 3GXE, 6NAJ

Gene	UniProt	Inclusion basis	Selected entries
ITGA5	P08648	matrix receptor	9B9J, 9P6S, 9B9K, 9DIA
ITGB1	P05556	matrix receptor	9NAB, 9B9J, 9P6S, 9B9K
ITGA1	P56199	matrix receptor	5HGJ, 1PT6, 4A0Q, 1QC5
ITGA2	P17301	matrix receptor	1V7P, 6NDH, 6ND9, 6ND8
ACAN	P16112	matrix component	4MD4, 7RDV, 9DFF, 9DFT
DCN	P07585	matrix component	None
LUM	P51884	matrix component	None
BGN	P21810	matrix component	None
LOX	P28300	matrix crosslinking	None
LOXL2	Q9Y4K0	matrix crosslinking	5ZE3
ELN	P15502	matrix component	None
MMP2	P08253	matrix remodelling	7XJO, 3AYU, 8H78, 1EAK
MMP3	P08254	matrix remodelling	1HY7, 1C3I, 4G9L, 1G49
MMP14	P50281	matrix remodelling	4P3C, 4P3D, 4QXU, 2MQS
TGFB1	P01137	matrix-associated signalling	8C7H, 8REW, 8VSD, 7Y1T
LTBP1	Q14766	latent TGF-beta context	9R3S
LTBP2	Q14767	matrix component	None
PIEZO1	Q92508	mechanosensing	8ZU3, 9VMX, 8YFC, 8YEZ
PTK2	Q05397	adhesion signalling	7PI4, 6YT6, 6YQ1, 3PXK
TLN1	Q9Y490	adhesion linkage	4DJ9, 1SYQ, 2MWN
COL1A1	P02452	matrix component	5CTD, 5CTI, 5CVA, 5CVB
COL1A2	P08123	matrix component	5CTD, 5CTI, 5CVA, 8YV3

Table S3. Complete 70-entry assembly-1 inventory. Selection ranks are within selecting genes. Pairs are retained single-accession panel chain pairs, not independent biological replicates. Full entry-specific mappings and retrieval URLs appear in Supplementary Data S3.

PDB	Selecting gene:rank	Processed residues	Retained pairs	Chimeric pairs excluded
13BF	COL6A3:1	487	0	0
1C3I	MMP3:2	343	1	0
1EAK	MMP2:4	847	1	0
1G49	MMP3:4	169	0	0
1HY7	MMP3:1	162	0	0
1PT6	ITGA1:2	192	0	0
1QC5	ITGA1:4	382	1	0
1SYQ	TLN1:2	284	0	0
1UZJ	FBN1:1	162	0	0
1V7P	ITGA2:1	454	0	0
21DU	ADGRG6:1	1039	0	0
2MQS	MMP14:4	301	0	0
2MWN	TLN1:3	117	0	0
2SEB	COL2A1:4	586	0	0
3AYU	MMP2:2	176	0	0
3FER	FLNB:2	234	0	0
3GXE	FN1:3	99	1	0
3PXK	PTK2:4	514	1	0
4A0Q	ITGA1:3	187	0	0
4B7L	FLNB:1	328	0	0
4DJ9	TLN1:1	264	0	0
4G9L	MMP3:3	168	0	0
4GYX	COL3A1:3	82	3	0
4MD4	ACAN:1	382	0	0
4P3C	MMP14:1	898	0	0
4P3D	MMP14:2	898	0	0
4QXU	MMP14:3	450	0	0

PDB	Selecting gene:rank	Processed residues	Retained pairs	Chimeric pairs excluded
5CTD	COL1A1:1, COL1A2:1	207	0	3
5CTI	COL1A1:2, COL1A2:2	208	0	3
5CVA	COL1A1:3, COL1A2:3	192	0	3
5CVB	COL1A1:4	193	0	3
5DCP	FLNB:3	164	0	0
5HGJ	ITGA1:1	194	0	0
5OCX	COL2A1:1	443	0	0
5ZE3	LOXL2:1	441	0	0
6NAJ	FN1:4	1699	0	0
6ND8	ITGA2:4	444	0	0
6ND9	ITGA2:3	444	0	0
6NDH	ITGA2:2	444	0	0
6NIX	COL2A1:3	380	0	0
6SNK	COL6A3:3	188	0	0
6YQ1	PTK2:3	269	0	0
6YT6	PTK2:2	256	0	0
7NWL	FN1:2	1747	3	0
7NZE	COL2A1:2	770	0	0
7PI4	PTK2:1	613	0	0
7RDV	ACAN:2	787	0	0
7WWR	COL3A1:2	90	3	0
7WWS	COL3A1:1	81	3	0
7XAN	COL3A1:4	78	3	0
7XJO	MMP2:1	169	0	0
7Y1T	TGFB1:4	1030	0	0
8C7H	TGFB1:1	1208	4	0
8H78	MMP2:3	168	0	0
8REW	TGFB1:2	1609	6	0
8VSD	TGFB1:3	2109	1	0
8YEZ	PIEZO1:4	3837	3	0
8YFC	PIEZO1:3	3903	3	0
8YV3	COL1A2:4	90	3	0
8ZU3	PIEZO1:1	3903	3	0
9B9J	ITGA5:1, ITGB1:2	1055	1	0
9B9K	ITGA5:3, ITGB1:4	1142	1	0
9DFF	ACAN:3	306	0	0
9DFT	ACAN:4	308	0	0
9DIA	ITGA5:4	883	1	0
9GTU	COL6A3:2	706	0	0
9NAB	ITGB1:1	1106	0	0
9P6S	FN1:1, ITGA5:2, ITGB1:3	1224	3	0
9R3S	LTBP1:1	723	2	0
9VMX	PIEZO1:2	3903	3	0

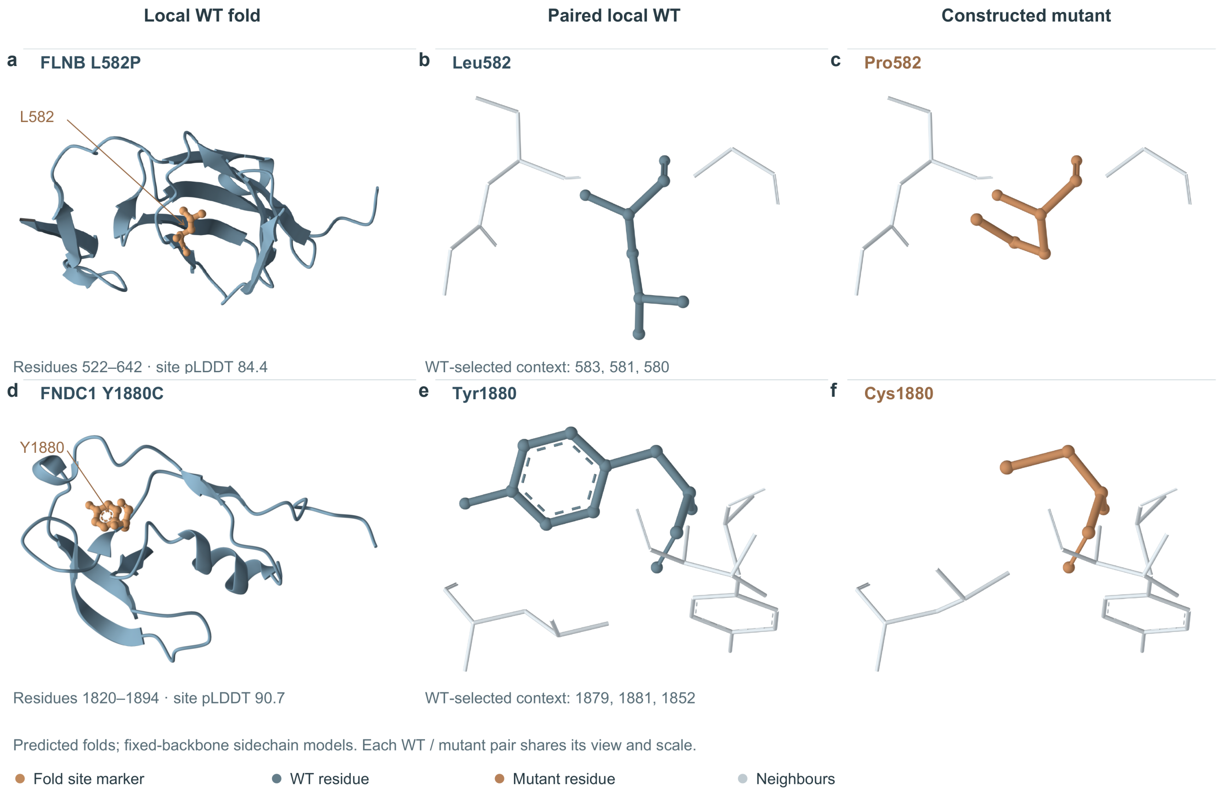

Figure S3. **Structural localization of candidates with discordant stability predictions.** Rows: FLNB L582P (**a**–**c**) and FNDC1 Y1880C (**d**–**f**). Left: local AlphaFold WT folds and labelled residues, with site pLDDT 84.4 and 90.7. Middle/right: paired locally optimized WT/EvoEF-built mutant sidechains, sharing camera and scale. Neighbours were selected within 4 Å in WT. L582P has a smaller DynaMut2 estimate ($0.219\,\mathrm{kcal\,mol^{-1}}$); Y1880C has an oppositely signed estimate ($-0.301\,\mathrm{kcal\,mol^{-1}}$) and changes threshold classification with the EvoEF WT reference. These panels preserve the original shortlist's structural record; they do not establish changed packing, stability or function. All four candidates and their scores remain in main Table 1.

References

[1] Erat, M.C.; Sladek, B.; Campbell, I.D.; Vakonakis, I. Structural analysis of collagen type I interactions with human fibronectin reveals a cooperative binding mode. *J. Biol. Chem.* **2013**, *288*, 17441–17450. doi:10.1074/jbc.M113.469841.
